# Supplementary material for: Dimensional reduction of emergent spatiotemporal cortical dynamics via a maximum entropy moment closure
Source: PLoS Comput Biol. 2020 Jun 9;16(6):e1007265. doi: 10.1371/journal.pcbi.1007265 (PMC7304648; doi:10.1371/journal.pcbi.1007265)
Supplement: S1 Appendix — (DOCX) [file pcbi.1007265.s001.docx]

**S1 Appendix A: Derivation of Fokker-Planck equation**

The kinetic equation Eq.(16) for the current-based I&F neuronal dynamics Eq.(1) in the main-text is a partial differential equation with a delay in $v$-space. If the voltage jump values induced by instantaneous synaptic currents $f_{j}^{Q}$, $S_{fast}^{QE}K_{fast}^{QE}\left( \left| c_{j}-c_{i} \right| \right)$ and $S_{fast}^{QI}K_{fast}^{QI}\left( \left| c_{j}-c_{i} \right| \right)$ are sufficiently small, we could Taylor-expand the difference terms in Eq.(16) and thus derive a diffusion equation as an approximation. Here we use external input $f_{j}^{Q}$ as an example. It is easy to show that to $\mathcal{O}\left( \left( f_{j}^{Q} \right)^{2} \right)$, the second term in Eq.(16) can be written as

$$\eta_{j}^{Q}\left[ \rho_{j}^{Q}\left( v-f_{j}^{Q},t \right)-\rho_{j}^{Q}\left( v,t \right) \right]$$

$$=\eta_{j}^{Q}\left[ \rho_{j}^{Q}\left( v,t \right)+\frac{\left( -f_{j}^{Q} \right)}{1}\partial_{v}\rho_{j}^{Q}\left( v,t \right)\left. +\frac{\left( -f_{j}^{Q} \right)^{2}}{2}\partial_{vv}\rho_{j}^{Q}\left( v,t \right)-\rho_{j}^{Q}\left( v,t \right) \right] \right.$$

$$\begin{aligned} =\eta_{j}^{Q}\partial_{v}\left[ \frac{\left( -f_{j}^{Q} \right)}{1}+\frac{\left( -f_{j}^{Q} \right)^{2}}{2}\partial_{v} \right]\rho_{j}^{Q}\left( v,t \right)+\mathcal{O}\left( \left( f_{j}^{Q} \right)^{3} \right) ,\#\left( A.1 \right) \end{aligned}$$

similar approximations for $S_{fast}^{QE}K_{fast}^{QE}\left( \left| c_{j}-c_{i} \right| \right)$,$S_{fast}^{QI}K_{fast}^{QI}\left( \left| c_{j}-c_{i} \right| \right)$. By substituting these terms, we could write the probability flux $J_{j}^{Q}\left[ \rho_{j}^{Q}\left( v,t \right) \right]$ in the form of

$$J_{j}^{Q}\left[ \rho_{j}^{Q}\left( v,t \right) \right]=-\left[ \left( v-\left( V_{R}g_{L}+ f_{j}^{Q}\eta_{j}^{Q}+N_{E}S_{fast}^{QE}\sum_{i} K_{fast}^{QE}\left( \left| c_{j}-c_{i} \right| \right)m_{i}^{E} \right. \right. \right.$$

$$\left. \left. -N_{I}S_{fast}^{QI}\sum_{i} K_{fast}^{QI}\left( \left| c_{j}-c_{i} \right| \right)m_{i}^{I}+I_{j,slow}^{QE} \right)/{g_{L}} \right)$$

$$+\left( \left( f_{j}^{Q} \right)^{2}\eta_{j}^{Q}+N_{E}\sum_{i} {\left( S_{fast}^{QE}K_{fast}^{QE}\left( \left| c_{j}-c_{i} \right| \right) \right)^{2}m}_{i}^{E} \right.$$

$$\begin{aligned} \left. \left. + N_{I}\sum_{i} \left( S_{fast}^{QI}K_{fast}^{QI}\left( \left| c_{j}-c_{i} \right| \right) \right)^{2}m_{i}^{I} \right)/\left( 2g_{L} \right)\partial_{v} \right]\rho_{j}^{Q}\left( v,t \right).\#\left( A.2 \right) \end{aligned}$$

We then decompose terms in Eq.(A.2) into drift and diffusion coefficients expressed by Eqs.(19,20), then replace and re-write the probability flux $J_{j}^{Q}\left[ \rho_{j}^{Q}\left( v,t \right) \right]$ in the form of Eq.(18). Finally, by substituting, together with the approximate form of flux, we yield the Fokker-Planck type equation in Eq.(17).
