# Supplementary material for: Dimensional reduction of emergent spatiotemporal cortical dynamics via a maximum entropy moment closure
Source: PLoS Comput Biol. 2020 Jun 9;16(6):e1007265. doi: 10.1371/journal.pcbi.1007265 (PMC7304648; doi:10.1371/journal.pcbi.1007265)
Supplement: S2 Appendix — (DOCX) [file pcbi.1007265.s002.docx]

**S2 Appendix B: Reduction of the slow current** $\boldsymbol{I}_{\boldsymbol{i}}^{\boldsymbol{s}}$**.**

Here we give a simple example of the reduction process for a slow post-synaptic input $I_{i}^{s}$ in voltage moment. The case can be described as,

$$\begin{aligned} \frac{d}{dt}V_{i}= -g_{L}\left( V_{i}-V_{R} \right)+I_{i}^{s},\#\left( B.1 \right) \end{aligned}$$

$$\begin{aligned} \sigma_{s}\frac{d}{dt}I_{i}^{s}= -I_{i}^{s}+\sum_{k} S_{i}^{s}\delta\left( t-T_{k} \right).\#\left( B.2 \right) \end{aligned}$$

We consider the probability density function

$$\begin{aligned} \rho\left( v,i^{s},t \right)= \frac{1}{N}\sum_{i=1}^{N} \mathbb{E}\left[ \delta\left( v-V_{i} \right)\delta\left( i^{s}-I_{i}^{s} \right) \right].\#\left( B.3 \right) \end{aligned}$$

First, we calculate $\rho\left( v,i^{s},t+\Delta t \right)$, here we use the identity of conditional probability to write

$$\mathbb{E}\left[ \delta\left( v-V_{i}\left( t+\Delta t \right) \right)\delta\left( i^{s}-I_{i}^{s}\left( t+\Delta t \right) \right) \right]$$

$$\begin{aligned} \mathbb{= E}\left[ \mathbb{E}\left[ \delta\left( v-V_{i}\left( t+\Delta t \right) \right)\delta\left( i^{s}-I_{i}^{s}\left( t+\Delta t \right) \right)|V_{i}\left( t \right),I_{i}^{s}\left( t \right) \right] \right].\#\left( B.4 \right) \end{aligned}$$

Given a neuron that has a voltage $V_{i}\left( t \right)$ and a slow current $I_{i}^{s}\left( t \right)$ at time $t$, there will be two possibilities in the interval $\left( t,t+\Delta t \right)$: 1) the neuron receives a spike with probability $\nu\left( t \right)\Delta t$ (for simplification, we use $\nu$ to represent the rate of the only one Poisson spike train $\left\{ T_{k} \right\}$ used in this example); 2) the neuron, with probability $\left( 1-\nu\left( t \right)\Delta t \right)$, receives no spikes. Therefore, to $\mathcal{O}\left( \Delta t \right)$, we have

$$\mathbb{E}\left[ \delta\left( v-V_{i}\left( t+\Delta t \right) \right)\delta\left( i^{s}-I_{i}^{s}\left( t+\Delta t \right) \right)|V_{i}\left( t \right),I_{i}^{s}\left( t \right) \right]$$

$$=\left( 1-\nu\left( t \right)\Delta t \right)\delta\left( v-\left[ V_{i}+\left[ -g_{L}\left( V_{i}-V_{R} \right)+I_{i}^{s} \right]\Delta t \right] \right)\delta\left( i^{s}-\left[ I_{i}^{s}-\frac{I_{i}^{s}\Delta t}{\sigma_{s}} \right] \right)$$

$$+\nu\left( t \right)\Delta t\delta\left( v-V_{i} \right)\delta\left( i^{s}-\left[ I_{i}^{s}+\frac{S_{i}^{s}}{\sigma_{s}} \right] \right)$$

$$= \left( 1-\nu\left( t \right)\Delta t \right)\left\{ \delta\left( v-V_{i} \right)+\frac{\partial}{\partial_{v}}\delta\left( v-V_{i} \right)\left[ g_{L}\left( V_{i}-V_{R} \right)-I_{i}^{s} \right]\Delta t \right\}$$

$$\begin{aligned} \times\delta\left( i^{s}-I_{i}^{s} \right)+\nu\left( t \right)\Delta t\delta\left( v-V_{i} \right)\delta\left( i^{s}-\left[ I_{i}^{s}+\frac{S_{i}^{s}}{\sigma_{s}} \right] \right).\#\left( B.5 \right) \end{aligned}$$

After considering the limitation $\sigma_{slow}\to\infty, \delta\left( x-a \right)a=\delta\left( x-a \right)x$and probability up to $\mathcal{O}\left( \Delta t \right)$, yields

$$\mathbb{E}\left[ \delta\left( v-V_{i}\left( t+\Delta t \right) \right)\delta\left( i^{s}-I_{i}^{s}\left( t+\Delta t \right) \right)|V_{i}\left( t \right),I_{i}^{s}\left( t \right) \right]$$

$$=\delta\left( v-V_{i} \right)\delta\left( i^{s}-I_{i}^{s} \right)$$

$$+\frac{\partial}{\partial_{v}}\left[ \delta\left( v-V_{i} \right)\delta\left( i^{s}-I_{i}^{s} \right)\left[ g_{L}\left( v-V_{R} \right)-i^{s} \right] \right]\Delta t$$

$$+\nu\left( t \right)\Delta t\delta\left( v-V_{i} \right)\left[ \delta\left( i^{s}-\left[ I_{i}^{s}+\frac{S_{i}^{s}}{\sigma_{s}} \right] \right)-\delta\left( i^{s}-I_{i}^{s} \right) \right]$$

$$= \delta\left( v-V_{i} \right)\delta\left( i^{s}-I_{i}^{s} \right)$$

$$\begin{aligned} +\frac{\partial}{\partial_{v}}\left[ \delta\left( v-V_{i} \right)\delta\left( i^{s}-I_{i}^{s} \right)\left[ g_{L}\left( v-V_{R} \right)-i^{s} \right] \right]\Delta t . \#\left( B.6 \right) \end{aligned}$$

After taking expectation over all possible conditions $\left\{ V_{i}\left( t \right),I_{i}^{s}\left( t \right) \right\}$ and averaging over all neurons, we finally have

$$\begin{aligned} \partial_{t}\rho= \frac{\partial}{\partial_{v}}\left[ \left( g_{L}\left( v-V_{R} \right)-i^{s} \right)\rho\right] .\#\left( B.7 \right) \end{aligned}$$

Finally, we concluded that, consider the slow dynamics over an infinite long time-scale, its effect on voltage state was computed as a slowly changed integral current and reflected in the drift coefficient only.
