# Supplementary material for: Dimensional reduction of emergent spatiotemporal cortical dynamics via a maximum entropy moment closure
Source: PLoS Comput Biol. 2020 Jun 9;16(6):e1007265. doi: 10.1371/journal.pcbi.1007265 (PMC7304648; doi:10.1371/journal.pcbi.1007265)
Supplement: S3 Appendix — (DOCX) [file pcbi.1007265.s003.docx]

**S3 Appendix C: Derivation of the stationary solution** $\boldsymbol{\rho}_{\boldsymbol{Eq}}$

We here consider the derivation of the stationary solution to the Fokker-Planck equation Eq.(17) with the absorbing boundary condition at the threshold and resetting boundary condition at the reset point.

Specifically, we consider the Fokker-Planck equation Eq.(17) and the probability flux Eq.(18), with the boundary conditions Eq.(21) and the direct relation between $m_{j}^{Q}\left( t \right)$ and $J_{j}^{Q}\left[ \rho_{j}^{Q}\left( v,t \right) \right]$ in Eq.(23). Denote the equilibrium firing rate by $\bar{m}_{j}^{Q}$ and the equilibrium probability distribution by $\rho_{j,Eq}^{Q}\left( v \right)$. Fixing $\mu_{j}^{Q}$ and $\sigma_{j}^{Q}$, we take the long time simulations such that the $\rho_{j}^{Q}(v,t)$ will not change with the time$t$. Thus, we can derive $\rho_{j,Eq}^{Q}\left( v \right)$ by integrating the stationary equation Eq.(17) over $[v,V_{T}]$ and using the boundary conditions, namely

$$\int_{v}^{V_{T}} \partial_{v}J_{j}^{Q}\left[ \rho_{j,Eq}^{Q}\left( v \right) \right]= J_{j}^{Q}\left[ \rho_{j,Eq}^{Q}\left( V_{T} \right) \right]-J_{j}^{Q}\left[ \rho_{j,Eq}^{Q}\left( v \right) \right]$$

$$\begin{aligned} =\frac{\bar{m}_{j}^{Q}}{g_{L}}+\left( v-\mu_{j}^{Q} \right)\rho_{j,Eq}^{Q}\left( v \right)+\frac{\left( \sigma_{j}^{Q} \right)^{2}}{2}\partial_{v}\rho_{j,Eq}^{Q}\left( v \right)=0 .\#\left( C.1 \right) \end{aligned}$$

Then, multiply with $\exp\left( \left( v-\mu_{j}^{Q} \right)/{\sigma_{j}^{Q}} \right)^{2}$, we obtain

$$\frac{2\bar{m}_{j}^{Q}}{g_{L}\left( \sigma_{j}^{Q} \right)^{2}}\exp\left( \frac{v-\mu_{j}^{Q}}{\sigma_{j}^{Q}} \right)^{2}=-\left[ \frac{2\left( v-\mu_{j}^{Q} \right)}{\left( \sigma_{j}^{Q} \right)^{2}}+\partial_{v} \right]\rho_{j,Eq}^{Q}\left( v \right)\exp\left( \frac{v-\mu_{j}^{Q}}{\sigma_{j}^{Q}} \right)^{2}$$

$$\begin{aligned} =-\partial_{v}\left( \rho_{j,Eq}^{Q}\left( v \right)\exp\left( \frac{v-\mu_{j}^{Q}}{\sigma_{j}^{Q}} \right)^{2} \right). \#\left( C.2 \right) \end{aligned}$$

Setting$s=\left( v-\mu_{j}^{Q} \right)/{\sigma_{j}^{Q}}$, changing the variable and integrating$s$ from $\left( \max\left\{ v,V_{R} \right\}-\mu_{j}^{Q} \right)/{\sigma_{j}^{Q}}$ to $\left( V_{T}-\mu_{j}^{Q} \right)/{\sigma_{j}^{Q}}$, we have the equilibrium probability distribution,

$$\begin{aligned} \rho_{j,Eq}^{Q}\left( v \right)=\frac{2\bar{m}_{j}^{Q}}{g_{L}\sigma_{j}^{Q}}\exp\left[ -\left( \frac{v-\mu_{j}^{Q}}{\sigma_{j}^{Q}} \right)^{2} \right]\int_{\left( max\left\{ v,V_{R} \right\}-\mu_{j}^{Q} \right)/{\sigma_{j}^{Q}}}^{\left( V_{T}-\mu_{j}^{Q} \right)/{\sigma_{j}^{Q}}} \exp\left( s^{2} \right)ds. \#\left( C.3 \right) \end{aligned}$$

We remark the value of the coefficient $2\bar{m}_{j}^{Q}/g_{L}\sigma_{j}^{Q}$ in Eq.(C.3) can be obtained by normalizing the equilibrium probability distribution$\rho_{j,Eq}^{Q}\left( v \right).$ After that, we can further get the value of the equilibrium firing rate $\bar{m}_{j}^{Q}$.
