# Supplementary material for: Dimensional reduction of emergent spatiotemporal cortical dynamics via a maximum entropy moment closure
Source: PLoS Comput Biol. 2020 Jun 9;16(6):e1007265. doi: 10.1371/journal.pcbi.1007265 (PMC7304648; doi:10.1371/journal.pcbi.1007265)
Supplement: S4 Appendix — (DOCX) [file pcbi.1007265.s004.docx]

**S4 Appendix D: The maximum Entropy approximation**

The basic idea behind the Maximum Entropy Approximation proposed in [27] is: If we only give a finite set of moments $\left\{ \chi_{j}^{Q,k}\left( t \right) \right\}$, $k=1,2,\ldots,M$ and the terms $\mu_{j}^{Q}$ and $\sigma_{j}^{Q}$, and consider the general definition of moments, there are many possible voltage distributions $\rho_{j}^{Q}\left( v,t \right)$ which are compatible with the entire information we have in, i.e.,$\left\{ \chi_{j}^{Q,k}\left( t \right) \right\}$, $\mu_{j}^{Q}$ and $\sigma_{j}^{Q}$. The implementation of Maximum Entropy allows us to, from amongst these possible dynamic voltage distributions, try and choose the one that most resembles the stationary state $\rho_{Eq}\left( v \right)$. Here the stationary distribution $\rho_{Eq}(v)$ can be considered as the steady state of Eq.(17) by fixing the $\mu_{j}^{Q}$ and $\sigma_{j}^{Q}$ terms from time$t$. Thus, one uses the formula Eq.(C.3) in Appendix C to obtain the equilibrium solution. In this situation, we can allow $\mu_{j}^{Q}$ and $\sigma_{j}^{Q}$ to change under the dynamics of Eq.(17), and finally expect $\rho(v,t)$ to chase $\rho_{Eq}\left( v \right)$ on a relatively fast time-scale.

To find how close any voltage distribution $\rho(v,t)$ is to the equilibrium distribution $\rho_{Eq}$, here we consider a classic maximum entropy method. We state $-h(p)$ is the Kullback-Leibler (KL) divergence of $\rho\left( v,t \right)$ with respect to $\rho_{Eq}\left( v \right)$ given by

$$\begin{aligned} h\left( \rho\right)= -\rho\left( v,t \right)\log\left( \frac{\rho\left( v,t \right)}{\rho_{Eq}\left( v \right)} \right), \#\left( D.1 \right) \end{aligned}$$

and the entropy function

$$\begin{aligned} H\left( \rho\right)=\int_{-\infty}^{V_{T}} h\left( \rho\right)dv,\#\left( D.2 \right) \end{aligned}$$

the time derivative satisfies

$$\begin{aligned} \partial_{t}h= -\partial_{t}\rho\log\left( \frac{\rho}{\rho_{Eq}} \right)-\partial_{t}\rho=\partial_{v}\left[ J \right]\left[ log\left( \frac{\rho}{\rho_{Eq}} \right)+1 \right].\#\left( D.3 \right) \end{aligned}$$

Thus, integrating we obtain

$$\int_{-\infty}^{V_{T}} \partial_{t}hdv= \frac{m}{g_{L}}\left[ \log\left( m/\bar{m} \right)-\log\left( {\rho\left( V_{R} \right)}/{\rho_{Eq}\left( V_{R} \right)} \right) \right]$$

$$\begin{aligned} +\int_{-\infty}^{V_{T}} \rho\frac{2}{\sigma^{2}}\left[ \left( v-\mu\right)+\frac{\sigma^{2}\partial_{v}\rho}{2\rho} \right]^{2}dv-\frac{\bar{m}}{g_{L}}\int_{V_{R}}^{V_{T}} \frac{2J}{\sigma^{2}\rho_{Eq}}dv .\#\left( D.4 \right) \end{aligned}$$

Here we use the fact that when $v<V_{R}$, $\partial_{v}\rho_{Eq}=-{2\left( v-u \right)\rho_{Eq}}/{\sigma^{2}}$, when $v\geq V_{R}$, $\partial_{v}\rho_{Eq}=-{2\left( v-u \right)\rho_{Eq}}/{\sigma^{2}}-{2\bar{m}}/\left( \sigma^{2}g_{L} \right)$. Thus, the integral is only guaranteed to increase with time when the single-neuron firing-probability is sufficiently small. Hence, we define the maximum entropy distribution by

$$\begin{aligned} \mathrm{maximize}H\left( \rho\right) = -\int_{-\infty}^{V_{T}} \rho\left( v,t \right)\log\left( \frac{\rho\left( v,t \right)}{\rho_{Eq}\left( v \right)} \right)dv , \\ subject to \chi^{k}\left( t \right)= \int_{-\infty}^{V_{T}} v^{k}\rho\left( v,t \right)dv.\#(D.5) \end{aligned}$$

We can rewrite the above optimization problem using a Lagrange function with a set of Lagrange multipliers $\boldsymbol{\lambda}=\left[ \lambda_{0}, \lambda_{1}, \ldots,\lambda_{M} \right]^{t}$as

$$\begin{aligned} \mathcal{L}\left( \rho,\lambda\right)=H\left( \rho\right)+ \sum_{k=0}^{M} \lambda_{k}\left( v^{k}\int_{-\infty}^{V_{T}} \rho\left( v,t \right)dv-\chi^{k}\left( t \right) \right) .\#\left( D.6 \right) \end{aligned}$$

Some alternative optimization methods can be applied to find the Lagrange multipliers. Based on general optimization theory, the necessary conditions for the probability distribution to be a maximizer for this ME problem is that, $\rho$ must satisfy all the constraints given by Eq.(D.5b), and the partial derivative of $\mathcal{L}\left( \rho,\lambda\right)$ in Eq.(D.6) with respect to $\rho$ should equal to zero, which means $\left( {\partial\mathcal{L}\left( \rho,\lambda\right)}/{\partial\rho} \right)\left( \delta\rho\right)=0$ for all $\delta\rho\neq0$. Finally, we get the classical solution for this optimization problem

$$\begin{aligned} \rho\left( v,t \right)=\rho_{Eq}\left( v \right)\exp\left( \sum_{k=0}^{M} \lambda_{k}v^{k}-1 \right) .\#\left( D.7 \right) \end{aligned}$$
