# Supplementary material for: Dimensional reduction of emergent spatiotemporal cortical dynamics via a maximum entropy moment closure
Source: PLoS Comput Biol. 2020 Jun 9;16(6):e1007265. doi: 10.1371/journal.pcbi.1007265 (PMC7304648; doi:10.1371/journal.pcbi.1007265)
Supplement: S1 Additional Evidence — (DOCX) [file pcbi.1007265.s005.docx]

**S5 Additional evidence (Additional supporting evidence for our Results)**

We find that not every numerical simulation reveals a similar type of motion-like spatiotemporal activity pattern under the Hikosaka LMI stimulus.

Note that in Jancke et al. (Nature 2004), they imaged the responses to a drawn-out stimulus at a speed of 32 deg/sec and another slower speed of 16 deg/sec, then linked the cortical spatiotemporal patterns with the object physical speeds. We hypothesize that the similarity between illusory motion activity induced by LMI stimulus paradigm and the response to physical motion stimulus has a sensitivity to a particular speed. This has been discussed and simulated with the large-scale integrate-and-fire model by Rangan et al. Here we test our CG model using square stimuli moving at different speeds, 20 (S1L Fig), 64 deg/sec. S2B Fig summarizes the spatiotemporal population responses to various stimuli (including moving square previously fed to our model with a speed at 32 deg/sec), and compares the trajectories of propagating wave under these stimuli with that induced by Hikosaka LMI stimulus (the curve is shifted along the time axis to make it easier to compare). Observation shows that trajectory under moving square at a speed of 32 deg/sec (blue solid line in S2B Fig) has nearly the same slope with that under LMI stimulus (green dash line in S2B Fig), whereas trajectories under moving square stimuli with much faster (64 deg/sec) or much slower (20 deg/sec) speed have steeper (black solid line in S2B Fig) or gentler (red solid line in S2B Fig) slope. The hypothesis can still be verified by using drawn-out stimuli with different speeds (S1I, O Fig and S2C, D Fig).

**S1 Fig. Response patterns of CG moment model under other stimulus paradigms and using other parameter sets.**

(L, M) Moving square stimulus moving at another speed, (L) visual input, which is identical to the previous one shown in Figure 5D except for a slower moving speed, red line denotes this slower moving speed of about ${20}^{^{\circ}}/sec$, blue dash-line represents the previous moving speed ${32}^{^{\circ}}/sec$, (M) membrane potential pattern of CG moment model. (I, O) The first type drawn-out stimulus, stimulus is drawn out to full bar length at another speed, (I) visual input, which is identical to the previous one shown in Figure 5G except for a slower drawn-out speed, red line denotes this slower speed of about ${20}^{^{\circ}}/sec$, (O) membrane potential pattern of CG moment model. (P, Q) Reversed drawn-out stimulus, (P) visual input, this stimulus paradigm is the same as Hikosaka LMI in the initial time period, but after 60 ms, it initiates from the right area and is drawn inward, (Q) membrane potential pattern of CG moment model. (R, S) Hikosaka LMI paradigm stimulates CG moment model with very strong local inhibition, (R) visual input (Hikosaka LMI stimulus paradigm), (S) membrane potential patterns of CG moment model with strong local inhibition, the new local inhibitory connections $S_{new}^{QI}= S_{old}^{QI}\times1E1\gg S^{QE}$. (T-V) Single bar stimulus, (T) visual input, (U) experimental VSD images of cat primary visual cortex, (V) membrane potential pattern of CG moment model (original).

The previous study revealed that one phenomenally important condition for the emergence of motion illusion is non-uniformly space-distributed, persistent NMDA voltage components. To further validate this facilitation in our moment model and verify that the NMDA-dependent priming effect discovered in previous large-scale I&F model still exists and plays a crucial role in the generation of the motion illusion phenomenon in our CG moment model, we expose our model to a stimulus called ‘Reversed Drawn-out’ stimulus. Spatiotemporal pattern of this stimulus is much similar to that of the second type drawn-out stimulus, that is, in the initial stage, a square stimulus pines on the left area, but after 60 ms, instead of extending from the square to the right terminal, the bar stimulus reversely extends inward from the right terminal and ultimately joints with the initial square stimulus (S1P Fig). The trajectory of the propagating wave (S2E Fig), which has a slightly positive slope rather than a negative one, also supports this.

**S2 Fig. Another spatiotemporal diagram and more results of simulation responses under different stimulus paradigms.**

(A) Response to Hikosaka LMI stimulus, the first line shows spatiotemporal diagrams of population-averaged membrane potential of excitatory subpopulation (left), inhibitory subpopulation (middle) and aggregated result (right), Left plot in the bottom is time courses of population-averaged membrane potentials (same conventions as in the bottom left plot of Figure 3F), right panel shows the wave position as a function of time (same conventions as in the bottom middle and right plots of Figure 3F), the velocity is 0.042 = (1.91 – 1.12)/ (109 – 90) (mm/ms). (B) Responses to moving square stimuli at three different speeds. The first line shows spatiotemporal diagrams of population-averaged membrane potential under moving square stimulus at a speed of ${64}^{^{\circ}}/sec$ (left), ${32}^{^{\circ}}/sec$ (middle, corresponding to Figure 5D-F), and ${20}^{^{\circ}}/sec$ (right, corresponding to S1L Fig), the bottom plot summarizes the temporal functions of wave position at a speed of ${64}^{^{\circ}}/sec$ (black), ${32}^{^{\circ}}/sec$ (blue), and ${20}^{^{\circ}}/sec$ (red), corresponding result under Hikosaka LMI (green dash-line) is plotted for comparison. (C, D) Results under the first type drawn-out stimuli, (C) stimulus is drawn-out at a speed of about ${32}^{^{\circ}}/sec$(corresponding to Figure 5G-I), the velocity of traveling wave is 0.049 = (1.91 – 1.12)/ (126 – 110) (mm/ms) (D) stimulus is drawn-out at a speed of about ${20}^{^{\circ}}/sec$(corresponding to S1I, O Fig), the velocity of traveling wave is 0.029 = (1.91 – 1.12)/ (149 – 122) (mm/ms). (E) Results under reversed drawn-out stimulus (corresponding to S1P, Q Fig). (F) Results under Hikosaka LMI stimulus, using CG moment model with strong inhibition (corresponding to S1R, S Fig). Detailed descriptions for each subplot in (C-F) are in the same conventions as in (A). Color bar and spatial scales are in the same conventions as in Figure 3F.

As we mentioned in the first validation simulation, our model is directly derived from I&F spiking neuron model and refers to Rangan et al’s previous work, therefore, our model inherits the appropriate parameter regime and physiologically plausible network architecture of their large-scale I&F model. We alter the network structure by increasing local inhibition to a considerable level and suffered from this strong and fast suppression, our CG moment model, under the same Hikosaka LMI stimulus, triggers spatiotemporal population activity patterns which are completely different from previous one. S1R, S Fig and S2F Fig show the scenario under this inhibition-dominated circumstance. In the first time interval (in S1S Fig, before $9\Delta$ ms), although the patches directly exposed to external visual stimuli still hold high activities, these responses are quickly suppressed by strong and fast (in our CG moment model, time constant of locally inhibitory synaptic current is set to be infinitely small, thus the suppression effect on model cortex is instantaneous), and nearly no high-amplitude activity can spread out. This low-amplitude population activity in the first time interval makes it much harder to build the crucial NMDA voltage components with spatial gradient, thus the disappearance of the priming effect makes it almost impossible to observe propagating wave under this condition. Spatiotemporal diagram of population activities under this condition, displayed in the top line S2F Fig, also confirm this. It shows moderate responses evoked by the square and bar stimulus seem to be separated, and the response to bar stimulus in the second time interval is much similar to the activity pattern under single bar stimulus (S1T-V Fig).
