# Supplementary figures and images for: Dimensional reduction of emergent spatiotemporal cortical dynamics via a maximum entropy moment closure
Source: PLoS Comput Biol. 2020 Jun 9;16(6):e1007265. doi: 10.1371/journal.pcbi.1007265 (PMC7304648; doi:10.1371/journal.pcbi.1007265)

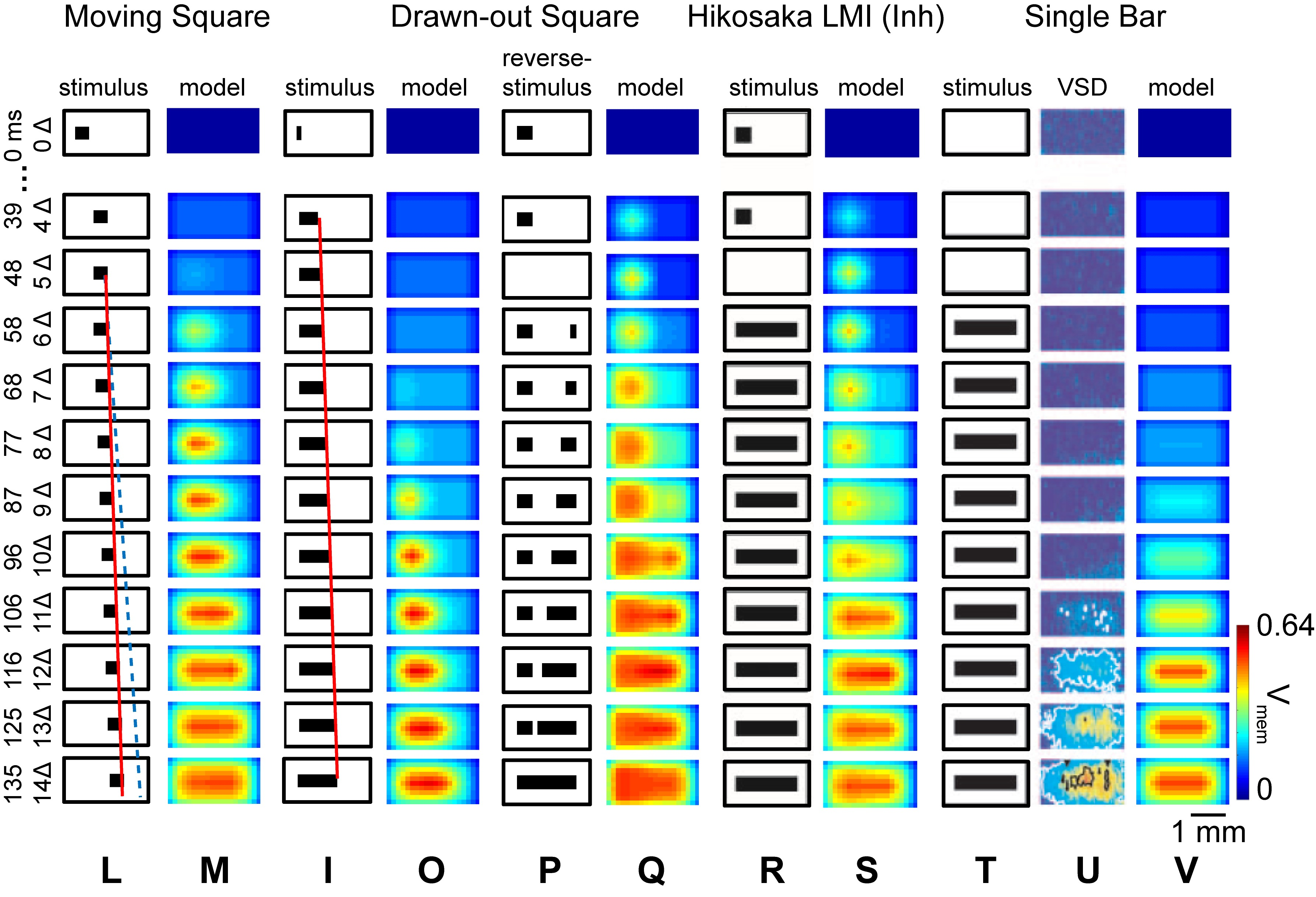

Supplement: S1 Fig — (L, M) Moving square stimulus moving at another speed, (L) visual input, which is identical to the previous one shown in Fig 5D except for a slower moving speed, red line denotes this slower moving speed of about 20°/sec, blue dash-line represents the previous moving speed 32°/sec, (M) membrane potential pattern of CG moment model. (I, O) The first type drawn-out stimulus, stimulus is drawn out to full bar length at another speed, (I) visual input, which is identical to the previous one shown in Fig 5G except for a slower drawn-out speed, red line denotes this slower speed of about 20°/sec, (O) membrane potential pattern of CG moment model. (P, Q) Reversed drawn-out stimulus, (P) visual input, this stimulus paradigm is the same as Hikosaka LMI in the initial time period, but after 60 ms, it initiates from the right area and is drawn inward, (Q) membrane potential pattern of CG moment model. (R, S) Hikosaka LMI paradigm stimulates CG moment model with very strong local inhibition, (R) visual input (Hikosaka LMI stimulus paradigm), (S) membrane potential patterns of CG moment model with strong local inhibition, the new local inhibitory connections SnewQI=SoldQI×1E1≫SQE. (T-V) Single bar stimulus, (T) visual input, (U) experimental VSD images of cat primary visual cortex, (V) membrane potential pattern of CG moment model (original). (TIF) [file pcbi.1007265.s006.tif]

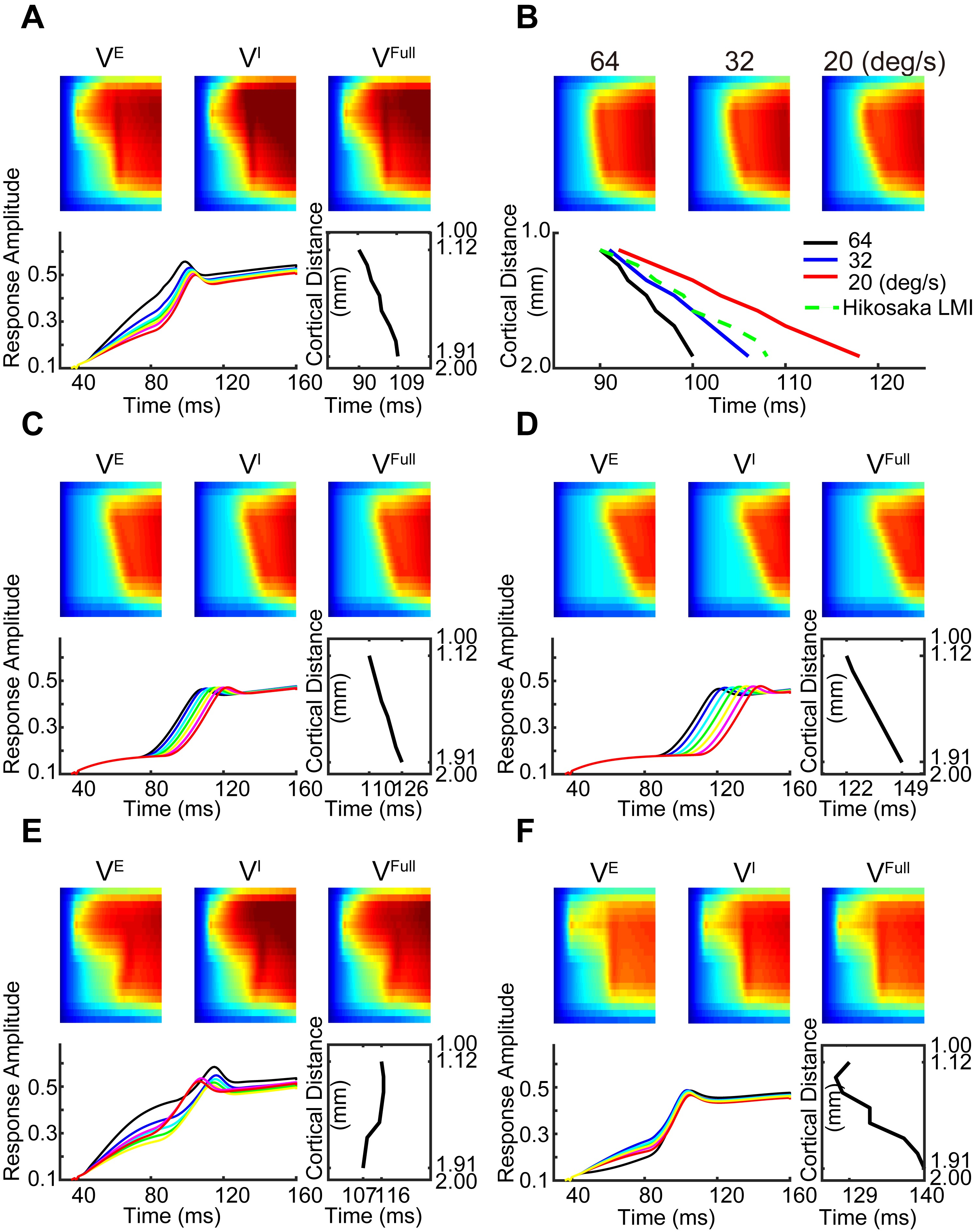

Supplement: S2 Fig — (A) Response to Hikosaka LMI stimulus, the first line shows spatiotemporal diagrams of population-averaged membrane potential of excitatory subpopulation (left), inhibitory subpopulation (middle) and aggregated result (right), Left plot in the bottom is time courses of population-averaged membrane potentials (same conventions as in the bottom left plot of Fig 3F), right panel shows the wave position as a function of time (same conventions as in the bottom middle and right plots of Fig 3F), the velocity is 0.042 = (1.91–1.12)/ (109–90) (mm/ms). (B) Responses to moving square stimuli at three different speeds. The first line shows spatiotemporal diagrams of population-averaged membrane potential under moving square stimulus at a speed of 64°/sec (left), 32°/sec (middle, corresponding to Fig 5D–5F), and 20°/sec (right, corresponding to S1L Fig), the bottom plot summarizes the temporal functions of wave position at a speed of 64°/sec (black), 32°/sec (blue), and 20°/sec (red), corresponding result under Hikosaka LMI (green dash-line) is plotted for comparison. (C, D) Results under the first type drawn-out stimuli, (C) stimulus is drawn-out at a speed of about 32°/sec (corresponding to Fig 5G–5I), the velocity of traveling wave is 0.049 = (1.91–1.12)/ (126–110) (mm/ms) (D) stimulus is drawn-out at a speed of about 20°/sec (corresponding to S1I and S1O Fig), the velocity of traveling wave is 0.029 = (1.91–1.12)/ (149–122) (mm/ms). (E) Results under reversed drawn-out stimulus (corresponding to S1P and S1Q Fig). (F) Results under Hikosaka LMI stimulus, using CG moment model with strong inhibition (corresponding to S1R and S1S Fig). Detailed descriptions for each subplot in (C-F) are in the same conventions as in (A). Color bar and spatial scales are in the same conventions as in Fig 3F. (TIF) [file pcbi.1007265.s007.tif]
