# Supplementary material for: Dimensional reduction of emergent spatiotemporal cortical dynamics via a maximum entropy moment closure
Source: PLoS Comput Biol. 2020 Jun 9;16(6):e1007265. doi: 10.1371/journal.pcbi.1007265 (PMC7304648; doi:10.1371/journal.pcbi.1007265)
Supplement: Attachment — Submitted filename: response_3_23.pdf [file pcbi.1007265.s009.pdf]

Dear Editor,

This is a revised version of the paper "Dimensional Reduction of Emergent Spatiotemporal Cortical Dynamics via a Maximum Entropy Moment Closure" (PCOMPBIOL-D-19-01160) written by Yuxiu Shao, Jiwei Zhang and Louis Tao.

We would like to thank the reviewers for their helpful suggestions and comments. We have modified the paper accordingly. An itemized list of responses is as follows.

Response to Reviewer 1.

- *It's fine.*

  Response: We thank the reviewer once again.

Response to Reviewer 2:

The authors have put considerable effort into clarification of their model, which is appreciated. However, they could still do a better job in places:

- *As far as I am able to see, they don't model individual neurons in this paper: no details are given of such a simulation and in the response to reviewer 3 they refer to other work. I find it confusing to refer to a large-scale V1 Model (l 348), and then to proceed by giving equations for individual neurons that suggest that a large-scale model of individual neurons has been implemented. The authors even state a number of neurons (l 170), but there is no evidence that for the purpose of this paper such a model was implemented. I propose that the authors state very clearly upfront that any modeling here is done at the population level, and that the individual neuron models only serve as a step towards the final population model, and that when they throw around numbers like 10^6 that they relate to a number of neurons that a comparable model would comprise if it were built.*
  I find it somewhat misleading to provide detailed descriptions of synaptic currents Eqs 2, 3, 6, 7, when it is clear from the Master equation that delta synapses are used (Eq. 15). The model clearly implements instantaneous transitions and does not contain any other dimension than the membrane potential. So, what is the point of a relatively lengthy section on individual neuron dynamics that is considerably simplified later on at the population level. For the purpose of this paper they could simply start off with delta synapses.

  Response: We apologize for this confusion. We did implement a large-scale network model, where the synaptic currents are as described. We are working with experimental collaborators to explain their recent data (and hope to be able to report on the results of the large-scale model soon). However, it is our goal to outline a possible framework for coarse-graining neuronal networks models in general, and in this manuscript, we focused on a coarse-grained, dimensional reduction of a large-scale network model of V1. In particular, the cortical phenomena we considered in this paper have time-scales that are longer than AMPA and GABA synapses but are shorter than NMDA synapses. Therefore, we have chosen to illustrate the coarse-graining procedure as applied explicitly to delta (AMPA and GABA) synapses. We have rewritten the following paragraphs to clarify:

——— Main text ———

The Feedforward LGN Input

Our V1 modeling starts with the LGN module. The LGN cells come in two polarities, "On-" and "Off-" cells, each V1 cell receives synaptic inputs from a collection of both On- and Off-LGN cells, with these two types of LGNs segregated spatially into 2D Gabor-like patterns. In the most detailed large-scale IF model, we randomly sample about $\mathcal{O}(10^1)$ LGN cells within the 2D Gabor envelope and connect them to the same V1 neuron. N_{lgn} LGN cells connected to the jth V1 neurons create a sequence of spikes at times $T^{Y}_{(i,k)}$, which is a Poisson process. Each spike causes a synaptic current of synaptic strength S^{QY}, so then the total external LGN input I^{QY}_j can be modeled as,

$$I^{QY}_j = S^{QY} \sum_{i=1}^{N_{lgn}} \sum_k \alpha_{ext} \left(t - T^{Y}_{i,k}\right) = S^{QY} \sum_{i=1}^{N_{lgn}} \sum_k \delta_{ext} \left(t - T^{Y}_{i,k}\right). \quad (2)$$

The temporal kernel α_{ext} is an α-function and models the time course of the synaptic current induced by each LGN spike (each $T^{Y}_{i,k}$) [60, 61]. In the following coarse-graining reduction, the time scale of α_{ext} is assumed to be infinitely fast and model each spike as a delta function, $\delta(t-T^{Y}_{i,k})$. (Here we are explicitly modeling phenomena slower than AMPA and GABA but faster than NMDA time-scales.) Our first coarse-graining approximation is to use continuous rate $\eta^{ext}_j(t)$ rather than discrete Poisson spikes $\delta(t-T^{Y}_{i,k})$ to describe the total external input into the jth V1 neuron arrives from N_{lgn} LGN cells [13, 62], so we let

$$I^{QY}_j = S^{QY} * \eta^{ext}_j \left(t\right). \quad (3)$$

According to different spatiotemporal patterns of visual stimuli, the continuous, time-dependent rate of the Poisson process $\eta^{ext}_j(t)$ varies.

For the rotating drifting grating stimulus used in the first experiment, the visual stimulus is an intensity pattern $I(\mathbf{X}, t)$ given by

$$I(\mathbf{X}, t) = I_0[1 + \epsilon \mathrm{sin}(\mathbf{kX} - \omega t + \varphi)], \quad (4)$$

where φ describes the stimulus spatial phase and $\mathbf{k} = |k|(\cos\theta_t, \sin\theta_t)$ reflects the spatial frequency and instantaneous orientation at time t. The response of each cell in the LGN module can be modeled as a rectified, linear spatiotemporal convolution of the visual stimuli, where spatial and temporal kernels are constrained by experiments[42, 59]. Then, following Shelley & McLaughlin [61], the total, continuous current input into the jth cortical neuron is approximated by

$$\eta^{ext,DG}_j \left(t\right) = C\epsilon \left[1 + \frac{1}{2}\left(1 + cos2\left(\theta_j - \theta_t\right)\right) sin(\omega t - (\varphi_j - \varphi))\right], \quad (5)$$

where θ_j reflects preferred orientation and φ_j the preferred phase of the jth cortical neuron. Note that, in the large-scale I&F model, the index j labels single neuron, but in the coarse-grained network model (see below), we assume the V1 neurons within one coarse-grained patch are homogeneous, so the index j can also index each individual neurons in the jth homogeneous patch.

In our second experiment, transient On- and Off-visual stimuli were used to probe the dynamics of the On- and Off-visual pathways. The On- and Off-visual pathways already exhibit differences at the LGN [63-66], such as spatially segregated On- and Off- afferent couplings from the RGCs, different response times of the On- and Off-visual pathways and

so on. Here we use an α-function to describe the total, temporal responses of a collection of LGN cells to transient On- and Off-stimuli. So, the continuous, external current input under this condition is

$$\eta_j^{ext,On(Off)}(t) = \frac{t}{\tau_1^2}\exp\left(-\frac{t}{\tau_1}\right) - \frac{t}{\tau_2^2}\exp\left(-\frac{t}{\tau_2}\right). \tag{6}$$

We use two sets of parameters (τ_1, τ_2 in Eq (6)) for $\eta_j^{(ext,On)}$and $\eta_j^{(ext,Off)}$ to model the On- and Off-LGN feedforward time courses, respectively (Fig 1B).

- *I agree that rate-based models get fast transients wrong, and that may be a good argument for using them. I don't see any evidence that this is the case in this particular model, which begs the question: why model an already complex network with complex methods when simpler ones suffice?*

- *I admire the thinking about the closure method, the idea behind it is much clearer now. However, I'm in two minds about whether I personally would adopt the proposed solution here. A solution of the full system of coupled Fokker-Planck equations is not particularly time consuming, and not hard to implement. Implementing the moment closure method would need serious extra work on top of that, which overall may not save time. On the other hand, an understanding of network dynamics in terms of a few dominant nodes is valuable, in particular when models like these will be part of larger networks. These considerations should not stand in the way of publication: the authors should make their case and take up will follow, or not.*

Response: We thank the reviewer for these comments. Indeed, rate-based models get fast transients wrong and numerical solutions to Fokker-Planck equations are not too time-consuming. We would like to provide a less *ad-hoc*, and perhaps more unified, way of coarse-graining, by examining a first principles approach to coarse-graining and dimensional reduction. At the same time, it is a major concern of our dimensional reduction to identify the possible low-dimensional system of dominant dynamic nodes in a complex network, without introducing too many simplifying assumptions. We have added this line of thinking and clarifying remarks to the Discussion:

While these reductions are effective descriptions of coherent population dynamics, they yield systems of partial differential equations that are not always easily amenable to analysis. Mean-field approximations replace the ensemble density with the expected value of the network state variables. Further improvements [3, 9] take the fluctuations of neuronal activity into consideration, by introducing *ad hoc* Gaussian noise terms. Recently, a Master equation formalism proposed by Boustani and Destexhe, allows for a 'mesoscopic' level description of population dynamics. Their model can be used beyond stability analysis, but needed to make use of a phenomenologically fitted f-I curve. Other theoretical studies have focused on the dimensional reduction of complex neuronal network exhibiting heterogeneous dynamics. In particular, various studies [16, 48-55] have developed models with conductance moments. This leads to an infinite hierarchy of moments, where the dynamics of lower-order conditional moments depend on higher-order moments, necessitating moment closure assumptions.

Here we use a different method for reduction [19] which was previously shown to be effective for homogeneous I&F networks. In this framework, we obtained a system of ODEs of voltage moments, where the lower-order moments do not depend on higher-order moments. However, at each moment in time, the population firing rate is needed to evolve the system of ODEs. And, in order to compute the population firing rates, we

need to compute the full probability distribution function, which cannot be constructed uniquely from a finite set of voltage moments. Therefore, we need a different closure scheme and we choose to use a maximum entropy assumption. In this paper, we extend and apply this formalism towards modeling and analyzing large-scale coherent cortical activity in V1. First, we focus on a single V1 orientation hypercolumn before using it to model an extended network that spans roughly 5×3 orientation hypercolumns in V1, an area that contains $O(10^5)$ neurons. On this scale, VSD imaging of cat V1 revealed coherent wave propagation that may underlie motion perception [26].

While systems of PDEs derived from various kinetic theories [16, 48, 49, 51, 52, 54, 55] are not time consuming to solve, there is a general hope that we can construct an theory of emergent network dynamics in terms of a few dominant, lower order models. Through a maximum entropy voltage moment closure, our dimensional reduction highlights the reduced-dimensional dynamics of the (subthreshold) membrane potential distribution and makes explicit its effects on the dynamics of other neuronal populations. Mathematically, this reduction can be applied to any one-dimensional density distribution. Therefore, we do not need the diffusion approximation (FP equations) per se and can incorporate the effects of finite-size synapses [16, 53-56] and other cellular effects, e.g., adaption and short-term depression [7-9]. However, we note that these moment equations simplify greatly in linear I&F networks (a case where all the coefficients of the ODE system can be computed analytically from system parameters). Furthermore, because of the time-scale separation between the fast AMPA and the slow NMDA synapses, asymptotically we can treat the effects of the NMDA synapses as slow currents, keeping our probability distribution function one-dimensional (see Appendix B).

- *I also appreciate the authors' ambition to create a larger, more complex network.*

  Response: Thank you. We hope to report results from the large-scale network model soon.

- *I recommend that they publish their source code.*

  Response: We have uploaded the codes to github: `//github.com/shaonannan/CGNetwork_MEPMoment`

- *I think the appendices should not be in supporting material, but in the main text (as appendices). They are important.*

  Response: We have reorganized the manuscript and put the appendices in the main text.

Response to Reviewer 3:

- *The method is explained better in the revised manuscript, thanks to the additional appendices. The appendices are currently supplied as separate docx files, which are hard to read and not displayed properly on non-windows systems. Also, they will likely no be typeset by the professional typesetters. I would like to encourage the authors to include these appendices in the main manuscript, so that they will show as properly typeset as a pdfs.*

  Response: We have reorganized the manuscript in the new version by putting the appendices in the main text.

- *In the appendix D there are some point unclear still. The authors use two notations, ρ_{st} and ρ_{eq}. I guess both should be identical.*

  Response: Yes, this is a typo. We have corrected it.

- *In line 92 the authors state the form of the stationary solution. This is specified in terms of an integral, involving only the drift term, noted $D^{(1)}$ here. The authors do not state the form of $D^{(1)}$, neither refer to an equation where it is defined. Also, it seems to me that the expression is incorrect. What the authors likely mean is the method in Section 5.2 of Risken, "The Fokker-Planck equation" $\rho_{st}(x)$ $e^{\phi(x)}$ and $\phi(x) = \int^x D^1(x')/D^2(x')dx'$, where D^1 is the drift and D^2 the diffusion coefficient.*

  Response: Yes, the formula with $D^{(1)}(x')$ we used did come from Section 5 of Risken's textbook. We originally expected to use this formula to explain why we use the maximum entropy principle to find the most probable dynamical trajectory. It seems that it is not necessary to introduce this notation in this context. Therefore, in the revised manuscript, we delete $D^{(1)}(x')$ and rewrite this part in Appendices C and D.

- *What remains unclear in this appendix is whether the authors use the stationary distribution with standard boundary conditions or whether they use the one that includes the absorbing boundary condition at the threshold and the re-insertion of the flux at reset. From the main text, it seems they use the latter. This should be clarified in the Appendix by referring back to section C of the Appendix.*

  Response: The stationary distribution is the one that includes the absorbing boundary condition at the threshold and the re-insertion of the flux at reset. In the revised Appendix C, we present the detailed derivation of the stationary solution given in Eq. (C.3).

- *It is unclear to me if the term "maximum entropy closure" is indeed justified for the method the authors use. This question arises, because the authors state in Appendix C in line 69 "Furthermore, we induce an equilibrium firing rate to solve the equilibrium probability distribution."*
  It is not clear how they define the equilibrium rate for a non-stationary Fokker-Planck equation.
  More fundamental, my question is this: Is the author's method identical to maximizing, at each time point, the Shannon entropy of the voltage distribution under the constraints that the moments obey the constraints stated in line 114 of the Appendix?
  If yes, this is not obvious to me from the given material.

  Response: The statement of "Furthermore, we induce an equilibrium firing rate to solve the equilibrium probability distribution" is misleading and incorrect. How exactly to construct the correct distribution is an important issue, especially considering that μ_j^Q and σ_j^Q are not fixed in time. In general, we expect $\rho_j^Q(v,t)$ to chase $\rho_{eq}(v,t)$ on a relatively fast time-scale. In previous work [27], in an idealized, homogeneous network, we checked how good is this approximation of using an "equilibrium" $\rho_{eq}(v,t)$ to approximate the real $\rho_j^Q(v,t)$. Indeed, even in the large-scale V1 network, with network inhomogeneities and diverse visual inputs, this "numerical" approximation is reasonable, as we found that our network simulations were well fitted by our coarse-grained solutions. We have also rewritten Appendix C and D for clarification.

- *In line 101 of the Appendix, the authors call $h(\rho)$ the density function. I think it would be helpful to state here that this is the Kullback-Leibler divergence.*

  Response: Changed.

- *Typos: period (.) missing after eq. 23 line 508 space too much in "point ." line 592: broken senstence: "We call this closed-form by the augmented ODE system. found that in the simulations we performed in this paper, good performance can be achieved with only 4 augmented variables ..."*

  Response: Corrected.

- *Have all data underlying the figures and results presented in the manuscript been provided?*
  Large-scale datasets should be made available via a public repository as described in the PLOS Computational Biology data availability policy, and numerical data that underlies graphs or summary statistics should be provided in spreadsheet form as supporting information.
  Reviewer #1: None
  Reviewer #2: Yes
  Reviewer #3: Yes
  PLOS authors have the option to publish the peer review history of their article (what does this mean?). If published, this will include your full peer review and any attached files. If you choose "no", your identity will remain anonymous but your review may still be made public.
  Do you want your identity to be public for this peer review? For information about this choice, including consent withdrawal, please see our Privacy Policy.
  Reviewer #1: No
  Reviewer #2: No
  Reviewer #3: No

We thanks again for the referees' useful suggestions and comments to improve the quality of the paper.

Yuxiu Shao, Jiwei Zhang, and Louis Tao
