# Supplementary material for: Dimensional reduction of emergent spatiotemporal cortical dynamics via a maximum entropy moment closure
Source: PLoS Comput Biol. 2020 Jun 9;16(6):e1007265. doi: 10.1371/journal.pcbi.1007265 (PMC7304648; doi:10.1371/journal.pcbi.1007265)
Supplement: Attachment — Submitted filename: ResponseLetter_20191128.docx [file pcbi.1007265.s008.docx]

(PCOMPBIOL-D-19-01160) - [EMID:4f412bba2c31867f]

Title: "Dimensional Reduction of Emergent Spatiotemporal Cortical Dynamics via a Maximum Entropy Moment Closure"

Authors: Yuxiu Shao, Jiwei Zhang, Louis Tao

We thank the referees very much for their insightful comments and helpful suggestions. We have carefully reviewed the questions and comments and have revised the manuscript accordingly. Please find below our point-by-point responses. The changes are highlighted **in blue** in the manuscript and we also uploaded an Appendix file for review. We hope that the revised version is now suitable for publication in PLOS Computational Biology.

Response to Reviewer # 1

I think this paper is fine. Perhaps they can give more details or cite the relevant reference from how they get from (15) to (16).

**Response:** We detailed the derivation in a newly added Appendix A., and also cited the relevant references (Cai et al., 2006; Nykamp and Tranchina, 2000; Rangan et al., 2008; Zhang et al., 2014a; Zhang et al., 2014b) in the main text.

------ TEXT ------

The details are in Appendix A, and some relevant references can be found in (Cai et al., 2006; Nykamp and Tranchina, 2000; Rangan et al., 2008; Zhang et al., 2014a; Zhang et al., 2014b).

------ APPENDIX ------

**Appendix A.**

The kinetic equation

$$\frac{d\rho_{j}^{Q}\left( v,t \right)}{dt}= g_{L}\partial_{v}\left[ \left( v-V_{L}-\frac{I_{j,slow}^{QE}\left( t \right)}{g_{L}} \right)\rho_{j}^{Q}\left( v,t \right) \right]$$

$$+\eta_{j}^{Q}\left[ \rho_{j}^{Q}\left( v-f_{j}^{Q},t \right)-\rho_{j}^{Q}\left( v,t \right) \right]$$

$$+N_{E}\sum_{i} m_{i}^{E}\left( t \right)\left[ \rho_{j}^{Q}\left( v-S_{fast}^{QE}K_{fast}^{QE}\left( \left| c_{j}-c_{i} \right| \right),t \right)-\rho_{j}^{Q}\left( v,t \right) \right]$$

$$\begin{aligned} \begin{aligned} + N_{I}\sum_{i} m_{i}^{I}\left( t \right)\left[ \rho_{j}^{Q}\left( v+S_{fast}^{QI}K_{fast}^{QI}\left( \left| c_{j}-c_{i} \right| \right),t \right)-\rho_{j}^{Q}\left( v,t \right) \right] \end{aligned}\#\left( 15 \right) \end{aligned}$$

for the current-based I&F neuronal dynamics

$$\begin{aligned} \begin{aligned} \frac{d}{dt}V_{j}^{Q}= -\frac{1}{\tau_{V}}\left( V_{j}^{Q}-V^{L} \right)+ I_{j}^{QY}+I_{j}^{QE}- I_{j}^{QI} \end{aligned}\#\left( 1 \right) \end{aligned}$$

is a partial differential equation with a delay in $v$-space. If the voltage jump values induced by instantaneous synaptic currents $f_{j}^{Q}$, $S_{fast}^{QE}K_{fast}^{QE}\left( \left| c_{j}-c_{i} \right| \right)$ and $S_{fast}^{QI}K_{fast}^{QI}\left( \left| c_{j}-c_{i} \right| \right)$ are sufficiently small, we could Taylor-expand the difference terms in $\mathrm{Eq}\left( 15 \right)$ and thus derive a diffusion equation as an approximation. Here we use external input $f_{j}^{Q}$ as an example. It is easy to show that to $\mathcal{O}\left( \left( f_{j}^{Q} \right)^{2} \right)$, the second term in $\mathrm{Eq}\left( 15 \right)$ can be written as

$$\eta_{j}^{Q}\left[ \rho_{j}^{Q}\left( v-f_{j}^{Q},t \right)-\rho_{j}^{Q}\left( v,t \right) \right]$$

$$=\eta_{j}^{Q}\left[ \rho_{j}^{Q}\left( v,t \right)+\frac{\left( -f_{j}^{Q} \right)}{1}\partial_{v}\rho_{j}^{Q}\left( v,t \right) \right.$$

$$\left. +\frac{\left( -f_{j}^{Q} \right)^{2}}{2}\partial_{vv}\rho_{j}^{Q}\left( v,t \right)-\rho_{j}^{Q}\left( v,t \right) \right]$$

$$\begin{aligned} \begin{aligned} =\eta_{j}^{Q}\partial_{v}\left[ \frac{\left( -f_{j}^{Q} \right)}{1}+\frac{\left( -f_{j}^{Q} \right)^{2}}{2}\partial_{v} \right]\rho_{j}^{Q}\left( v,t \right)+\mathcal{O}\left( \left( f_{j}^{Q} \right)^{3} \right) \end{aligned}\#\left( 30 \right) \end{aligned}$$

and similar approximations for $S_{fast}^{QE}K_{fast}^{QE}\left( \left| c_{j}-c_{i} \right| \right)$ and $S_{fast}^{QI}K_{fast}^{QI}\left( \left| c_{j}-c_{i} \right| \right)$. By substituting these terms, we could write the probability flux $J_{j}^{Q}\left[ \rho_{j}^{Q}\left( v,t \right) \right]$ in the form of,

$$J_{j}^{Q}\left[ \rho_{j}^{Q}\left( v,t \right) \right]=-\left[ \left( v-\left( V_{L}g_{L}+ f_{j}^{Q}\eta_{j}^{Q}+N_{E}S_{fast}^{QE}\sum_{i} K_{fast}^{QE}\left( \left| c_{j}-c_{i} \right| \right)m_{i}^{E} \right. \right. \right.$$

$$\left. \left. -N_{I}S_{fast}^{QI}\sum_{i} K_{fast}^{QI}\left( \left| c_{j}-c_{i} \right| \right)m_{i}^{I}+I_{j,slow}^{QE} \right)/{g_{L}} \right)$$

$$+\left( \left( f_{j}^{Q} \right)^{2}\eta_{j}^{Q}+N_{E}\sum_{i} {\left( S_{fast}^{QE}K_{fast}^{QE}\left( \left| c_{j}-c_{i} \right| \right) \right)^{2}m}_{i}^{E} \right.$$

$$\begin{aligned} \begin{aligned} \left. \left. + N_{I}\sum_{i} \left( S_{fast}^{QI}K_{fast}^{QI}\left( \left| c_{j}-c_{i} \right| \right) \right)^{2}m_{i}^{I} \right)/\left( 2g_{L} \right)\partial_{v} \right]\rho_{j}^{Q}\left( v,t \right) \end{aligned}\#\left( 31 \right) \end{aligned}$$

we then decompose terms in $\mathrm{Eq}\left( 31 \right)$ into drift and diffusion coefficients,

$$\mu_{j}^{Q}=\left( V_{L}g_{L}+ f_{j}^{Q}\eta_{j}^{Q}+N_{E}S_{fast}^{QE}\sum_{i} K_{fast}^{QE}\left( \left| c_{j}-c_{i} \right| \right)m_{i}^{E} \right.$$

$$\begin{aligned} \begin{aligned} \left. -N_{I}S_{fast}^{QI}\sum_{i} K_{fast}^{QI}\left( \left| c_{j}-c_{i} \right| \right)m_{i}^{I}+I_{j,slow}^{QE} \right)/{g_{L}} \end{aligned}\#\left( 18 \right) \end{aligned}$$

$$\left( \sigma_{j}^{Q} \right)^{2}=\left( \left( f_{j}^{Q} \right)^{2}\eta_{j}^{Q}+N_{E}\sum_{i} {\left( S_{fast}^{QE}K_{fast}^{QE}\left( \left| c_{j}-c_{i} \right| \right) \right)^{2}m}_{i}^{E} \right.$$

$$\begin{aligned} \begin{aligned} \left. + N_{I}\sum_{i} \left( S_{fast}^{QI}K_{fast}^{QI}\left( \left| c_{j}-c_{i} \right| \right) \right)^{2}m_{i}^{I} \right)/{g_{L}} \end{aligned}\#\left( 19 \right) \end{aligned}$$

and thus replace and re-write the probability flux $J_{j}^{Q}\left[ \rho_{j}^{Q}\left( v,t \right) \right]$ of form

$$\begin{aligned} \begin{aligned} J_{j}^{Q}\left[ \rho_{j}^{Q}\left( v,t \right) \right]= -\left( v-\mu_{j}^{Q} \right)\rho_{j}^{Q}\left( v,t \right)-\frac{\left( \sigma_{j}^{Q} \right)^{2}}{2}\partial_{v}\rho_{j}^{Q}\left( v,t \right) \end{aligned}\#\left( 17 \right) \end{aligned}$$

Then, by substituting, together with the approximate form of flux, we yield the Fokker-Planck type equation in $\mathrm{Eq}\left( 16 \right)$.

------

Response to Reviewer # 2

The authors model published VSD data in a local network of V1 populations, using a reduced population density model obtained by moment closure.

1. One problem is that although the model replicates the data, the data appear not that challenging to model. The authors provide a plausible interpretation of the experimental data and provide a reasonable interpretation of the neural circuits that can generate them. But overall they seem more concerned with the internal workings of their model than with the data. Could these data have been equally well modeled with Wilson-Cowan dynamics? I’d expect so, and it would have been a relatively straightforward exercise for them to have tried this, after putting in the work to model the architecture. If that is the case, what is the value of their method for this model?

I’m surprised that earlier work on moment closure was not cited, e.g. (Ly & Tranchina, 2007). How does the authors’ work differ from that?

The authors simply refer to their earlier paper for details on their method and while I understand that they don’t want to repeat the full analysis here, there are certain non-trivial elements to this approximation that should have been discussed here. The introduction of fast and slow synaptic currents (NMDA times, line 179-180) would suggest a non trivial exercise because this would lead to a two dimensional density at least (membrane potential and conductance state). However, the Fokker-Planck equation is simply one dimensional (Eq. 16) and appears to be the standard population density equation. Does the closure method work on a 1D density?

Why do the authors assume synaptic efficacies are small? There is no need to rely on that assumption: Nykamp & Tranchina (2000), de Kamps (2003), Iyer et al (2013) give efficient algorithms for finite-size synapses. Experimental evidence that the diffusion approximation is warranted has not been presented by the authors. Is the moment closure method critically dependent on it?

**Response:** Let us address these related questions in a single response. First, we agree with the reviewer that Wilson-Cowan type models can be used to capture the experimentally observed cortical activities induced by illusory stimuli. Many phenomenological models, such as Wilson-Cowan, can be fitted to individual experiments. However, it is our goal to derive a single, unified model of large-scale neuronal networks, to avoid having to re-tune model parameters to fit each individual experiment. Furthermore, here we were able to incorporate higher-order (beyond second-order) dynamics and thus capture complicated subthreshold dynamics not present in Wilson-Cowan type of models.

As for the dimensional reduction of complex neuronal network exhibiting heterogeneous dynamics, many theoretical methods have been tried. In particular, various people, including Ly & Tranchina (Cai et al., 2006; Haskell et al., 2001; Ly and Tranchina, 2007; Rangan et al., 2008), have developed models with *conductance* moments. In many cases, this has led to an infinite hierarchy of moments, where the dynamics of lower-order conditional moments depend on higher-order moments, necessitating moment closure assumptions to reduce to a single PDE for the marginal probability distribution in V.

Here we use a different method for reduction (Zhang et al., 2014b) which was previously shown to be effective for homogeneous I&F networks. In this framework, we obtained a system of ODES of *voltage* moments, where the lower-order moments do **not** depend on higher-order moments. However, at each moment in time, the population firing rate is needed to evolve this system of ODES. And, in order to compute the population firing rates, we needed the full probability distribution function, which cannot be constructed uniquely from a finite set of voltage moments. Therefore, in place of the usual moment closure, a different closure is required, and we chose to use a maximum entropy assumption.

This reduction, in principle, can be applied to any one-dimensional density distribution. Therefore, we do not need the diffusion approximation *per se* and can incorporate the effects of finite-size synapses among others. However, we note that these moment equations simplify greatly in linear I&F networks when we can use the diffusion approximation (a case where all the coefficients of the ODE system can be computed analytically from system parameters). Furthermore, because of the time-scale separation between the fast AMPA and the slow NMDA synapses, asymptotically we can treat the effects of the NMDA synapses as slow currents, keeping our probability distribution function one-dimensional (see a newly added Appendix B for details).

We note that there is a tradeoff between more faithful models and numerical efficacy/analytical simplicity and here we have made modeling choices for analytical simplicity.

In light of these issues, in the manuscript, we have added the following text (Materials and Methods, Discussion) and an Appendix B for clarification. We thank again for the useful suggestions and comments.

------ TEXT ------

**Materials and Methods**

We first focus on the dynamic evolution of voltage probability distribution induced by various synaptic inputs. Therefore, we define the probability flux $J_{j}^{Q}\left[ \rho_{j}^{Q}\left( v,t \right) \right]$, i.e., the probability crossing voltage $v$ at time $t$. In $\mathrm{Eq}\left( 15 \right)$, there are two types of fluxes, one due to the relaxation dynamics and another induced by voltage jumps upon receiving a spike. Various researchers have utilized this type of Master equation to study analytically or numerically the evolution of voltage probability density and the network dynamics (e.g., (De Kamps, 2003; Ly, 2013; Ly and Tranchina, 2007; Nykamp and Tranchina, 2000)).

For further simplification, we assume that the voltage jumps due to the external Poisson spikes as well as the recurrent network spikes are small, so the Master equation $\mathrm{Eq}\left( 15 \right)$ can be approximated by a Fokker-Planck type equation,

$$\begin{aligned} \begin{aligned} \frac{d\rho_{j}^{Q}\left( v,t \right)}{dt}+g_{L}\partial_{v}J_{j}^{Q}\left[ \rho_{j}^{Q}\left( v,t \right) \right]=0，\mathrm{for}v\in\left[ V_{R},\left. V_{T} \right) \right. \end{aligned}\#\left( 16 \right) \end{aligned}$$

where the probability flux $J_{j}^{Q}\left[ \rho_{j}^{Q}\left( v,t \right) \right]$ is

$$\begin{aligned} \begin{aligned} J_{j}^{Q}\left[ \rho_{j}^{Q}\left( v,t \right) \right]= -\left( v-\mu_{j}^{Q} \right)\rho_{j}^{Q}\left( v,t \right)- \frac{\left( \sigma_{j}^{Q} \right)^{2}}{2}\partial_{v}\rho_{j}^{Q}\left( v,t \right) \end{aligned}\#\left( 17 \right) \end{aligned}$$

with drift and diffusion terms.

**Discussion**

Many theoretical studies have focused on the dimensional reduction of complex neuronal network exhibiting heterogeneous dynamics. In particular, various studies (Cai et al., 2006; Cai et al., 2004; Haskell et al., 2001; Ly, 2013; Ly and Tranchina, 2007; Mattia et al., 2019; Mattia and Giudice, 2002; Nykamp and Tranchina, 2000; Rangan et al., 2008) have developed models with conductance moments. This leads to an infinite hierarchy of moments, where the dynamics of lower-order conditional moments depend on higher-order moments, necessitating moment closure assumptions.

Here we use a different method for reduction (Zhang et al., 2014b) which was previously shown to be effective for homogeneous I&F networks. In this framework, we obtained a system of ODES of voltage moments, where the lower-order moments do not depend on higher-order moments. However, at each moment in time, the population firing rate is needed to evolve the system of ODEs. And, in order to compute the population firing rates, we need to compute the full probability distribution function, which cannot be constructed uniquely from a finite set of voltage moments. Therefore, we need a different closure scheme and we chose to use a maximum entropy assumption. In this paper, we extend and apply this formalism towards modeling and analyzing large-scale coherent cortical activity in V1. First, we focus on a single V1 orientation hypercolumn before using it to model an extended network that spans roughly $5\times3$ orientation hypercolumns in V1, an area that contains ~$\mathcal{O}\left( {10}^{5} \right)$ neurons. On this scale, VSD imaging of cat V1 revealed coherent wave propagation that may underlie motion perception (Rekauzke et al., 2016).

Mathematically, this reduction can be applied to any one-dimensional density distribution. Therefore, we do not need the diffusion approximation (FP equations) *per se* and can incorporate the effects of finite-size synapses (De Kamps, 2003; Ly, 2013; Mattia et al., 2019; Mattia and Giudice, 2002; Nykamp and Tranchina, 2000) and other cellular effects, e.g., adaption and short-term depression (Deco and Rolls, 2005; Fusi, 2003; Wong and Wang, 2006). However, we note that these moment equations simplify greatly in linear I&F networks (a case where all the coefficients of the ODE system can be computed analytically from system parameters). Furthermore, because of the time-scale separation between the fast AMPA and the slow NMDA synapses, asymptotically we can treat the effects of the NMDA synapses as slow currents, keeping our probability distribution function one-dimensional (see Appendix B.).

------ APPENDIX ------

**Appendix B.**

Here we give a simple example of the reduction process for a slow post-synaptic input $I_{i}^{s}$ in voltage moment.

$$\begin{aligned} \begin{aligned} \frac{d}{dt}V_{i}= -g_{L}\left( V_{i}-V_{R} \right)+I_{i}^{s} \end{aligned}\#\left( 32 \right) \end{aligned}$$

$$\begin{aligned} \begin{aligned} \sigma_{s}\frac{d}{dt}I_{i}^{s}= -I_{i}^{s}+\sum_{k} S_{i}^{s}\delta\left( t-T_{k} \right) \end{aligned}\#\left( 33 \right) \end{aligned}$$

we then consider the probability density function,

$$\begin{aligned} \begin{aligned} \rho\left( v,i^{s},t \right)= \frac{1}{N}\sum_{i=1}^{N} \mathbb{E}\left[ \delta\left( v-V_{i} \right)\delta\left( i^{s}-I_{i}^{s} \right) \right] \end{aligned}\#\left( 34 \right) \end{aligned}$$

first we calculate $\rho\left( v,i^{s},t+\Delta t \right)$, here we use the identity of conditional probability to write

$$\mathbb{E}\left[ \delta\left( v-V_{i}\left( t+\Delta t \right) \right)\delta\left( i^{s}-I_{i}^{s}\left( t+\Delta t \right) \right) \right]$$

$$\begin{aligned} \begin{aligned} \mathbb{= E}\left[ \mathbb{E}\left[ \delta\left( v-V_{i}\left( t+\Delta t \right) \right)\delta\left( i^{s}-I_{i}^{s}\left( t+\Delta t \right) \right)|V_{i}\left( t \right),I_{i}^{s}\left( t \right) \right] \right] \end{aligned}\#\left( 35 \right) \end{aligned}$$

Given a neuron that has a voltage $V_{i}\left( t \right)$ and a slow current $I_{i}^{s}\left( t \right)$ at time $t$, there will be two possibilities in the interval $\left( t,t+\Delta t \right)$: 1) the neuron receives a spike with probability $\nu\left( t \right)\Delta t$ (for simplification, we use $\nu$ to represent the rate of the only one Poisson spike train $\left\{ T_{k} \right\}$ used in this example); 2) the neuron, with probability $\left( 1-\nu\left( t \right)\Delta t \right)$, receives no spikes. Therefore, to $\mathcal{O}\left( \Delta t \right)$, we have

$$\mathbb{E}\left[ \delta\left( v-V_{i}\left( t+\Delta t \right) \right)\delta\left( i^{s}-I_{i}^{s}\left( t+\Delta t \right) \right)|V_{i}\left( t \right),I_{i}^{s}\left( t \right) \right]$$

$$=\left( 1-\nu\left( t \right)\Delta t \right)\delta\left( v-\left[ V_{i}+\left[ -g_{L}\left( V_{i}-V_{R} \right)+I_{i}^{s} \right]\Delta t \right] \right)\delta\left( i^{s}-\left[ I_{i}^{s}-\frac{I_{i}^{s}\Delta t}{\sigma_{s}} \right] \right)$$

$$+\nu\left( t \right)\Delta t\delta\left( v-V_{i} \right)\delta\left( i^{s}-\left[ I_{i}^{s}+\frac{S_{i}^{s}}{\sigma_{s}} \right] \right)$$

$$= \left( 1-\nu\left( t \right)\Delta t \right)\left\{ \delta\left( v-V_{i} \right)+\frac{\partial}{\partial_{v}}\delta\left( v-V_{i} \right)\left[ g_{L}\left( V_{i}-V_{R} \right)-I_{i}^{s} \right]\Delta t \right\}$$

$$\begin{aligned} \begin{aligned} \times\delta\left( i^{s}-I_{i}^{s} \right)+\nu\left( t \right)\Delta t\delta\left( v-V_{i} \right)\delta\left( i^{s}-\left[ I_{i}^{s}+\frac{S_{i}^{s}}{\sigma_{s}} \right] \right) \end{aligned}\#\left( 36 \right) \end{aligned}$$

After considering the limitation $\sigma_{slow}\to\infty, \delta\left( x-a \right)a=\delta\left( x-a \right)x$and probability up to $\mathcal{O}\left( \Delta t \right)$, yields

$$\mathbb{E}\left[ \delta\left( v-V_{i}\left( t+\Delta t \right) \right)\delta\left( i^{s}-I_{i}^{s}\left( t+\Delta t \right) \right)|V_{i}\left( t \right),I_{i}^{s}\left( t \right) \right]$$

$$=\delta\left( v-V_{i} \right)\delta\left( i^{s}-I_{i}^{s} \right)$$

$$+\frac{\partial}{\partial_{v}}\left[ \delta\left( v-V_{i} \right)\delta\left( i^{s}-I_{i}^{s} \right)\left[ g_{L}\left( v-V_{R} \right)-i^{s} \right] \right]\Delta t$$

$$+\nu\left( t \right)\Delta t\delta\left( v-V_{i} \right)\left[ \delta\left( i^{s}-\left[ I_{i}^{s}+\frac{S_{i}^{s}}{\sigma_{s}} \right] \right)-\delta\left( i^{s}-I_{i}^{s} \right) \right]$$

$$= \delta\left( v-V_{i} \right)\delta\left( i^{s}-I_{i}^{s} \right)$$

$$\begin{aligned} \begin{aligned} +\frac{\partial}{\partial_{v}}\left[ \delta\left( v-V_{i} \right)\delta\left( i^{s}-I_{i}^{s} \right)\left[ g_{L}\left( v-V_{R} \right)-i^{s} \right] \right]\Delta t \end{aligned}\#\left( 37 \right) \end{aligned}$$

After taking expectation over all possible conditions $\left\{ V_{i}\left( t \right),I_{i}^{s}\left( t \right) \right\}$ and averaging over all neurons, we finally have

$$\begin{aligned} \begin{aligned} \partial_{t}\rho= \frac{\partial}{\partial_{v}}\left[ \left( g_{L}\left( v-V_{R} \right)-i^{s} \right)\rho\right] \end{aligned}\#\left( 38 \right) \end{aligned}$$

Finally, we concluded that, consider the slow dynamics over an infinite long time-scale, its effect on voltage state was computed as a slowly changed integral current and reflected in the drift coefficient only.

------

2. The authors state that two to four moments are sufficient to represent the density. This suggests that the density is never far from equilibrium, in particular if only two moments suffice. The authors could have implemented the network using the Fokker-Planck equation and compare the density spanned by the two (or four) moments with that of the FP equation. This would have shown something about how well their method performs.

**Response:** Thank you for this helpful suggestion. In our previous paper (Zhang et al., 2019), we did compare some of the important statistical dynamical features, e.g., first order statistics (firing rates and voltage distribution) and high-order statistics (such as multiple firing events and synchrony), between the LIF network, and the corresponding Masters and FP equations. For the comparison of densities, one can see Figure 3 in (Zhang et al., 2019). (Figure is included below.) For further comparison and performance of our reduction, please refer to the following (Zhang and Rangan, 2015; Zhang et al., 2019), in which we compare in detail the Master equation or FP equation with our moment closure method in simulating a variety of dynamical regimes, including homogeneous and synchronous network activities. For homogeneous networks activity, our reduction can capture the firing rate and density distribution almost exactly, and for synchronous network activity, it can faithfully capture highly heterogeneous neuronal network dynamics such as multiple firing events (or MFEs; please see Figure 4 in (Zhang and Rangan, 2015), also included below).

Figure 3 in (Zhang et al., 2019) A nearly homogeneous scenario for our PEA-framework. In panels-A and B we compare the steady-state voltage distributions for $\rho^{E}\left( v \right)$ and $\rho^{I}\left( v \right)$, respectively. The red line is achieved by IF model and the green dash line by the Master equation. In panels C and D we compare the steady-state voltage distributions for $\rho^{E}\left( v \right)$ and $\rho^{E}\left( v \right)$ respectively. The red line is achieved by IF equation and the blue line by Fokker-Planck equation.

Figure 4 in (Zhang and Rangan, 2015) Panel-a illustrates the dynamics of this IF-network; Panel-b and Panel-c are similar to Panel-a, except that they illustrate the system-trajectories corresponding to master equation and our moment model (derive from FP equation), respectively. The feedforward input to this regime is of higher rate and lower strength, and thus is more like a mean current input. This input, in conjunction with a stronger recurrent excitation and weaker recurrent inhibition (differ from the parameter regime in the condition of motion illusion), causes this regime to generate MFEs more frequently; this regime generates frequent large MFEs with a characteristic frequency close to 32 Hz.

3. The authors are correct that a well motivated reduction of FP equations to ODEs is computationally efficient for large networks and worthwhile pursuing. To make that case, in my opinion, they should have modeled a network that is not described well by rate based equations (Wilson-Cowan), but that is by FP (or other population based methods) as well as by their method.

**Response:** Rate-based models can be tuned or fitted to match experimentally observed networks activities. But phenomenologically-based rate-based models cannot capture the rapid, transient changes seen in spiking neuronal network model simulations (Gerstner, 2000). In previous work, we showed that a hybrid framework can capture both the transient synchronous firing of many neurons (which we called multiple-firing-events or MFEs; (Zhang et al., 2014a; Zhang and Rangan, 2015; Zhang et al., 2019; Zhang et al., 2014b)) and population activity well described by the usual ensemble averages. This hybrid formalism, the partitioned ensemble average, treats the two problems separately and considers the periods of fast synchronous spiking as special transitions between coarse-grained states well-described by usual population density methods (e.g., a Masters or a Fokker-Planck description) (Cai et al., 2006; Rangan et al., 2008).

In the present case, we have checked that our large-scale I&F network model, in reproducing the essential dynamic features of motion illusion, is in a dynamical regime where there are few or no MFEs. In the line-motion illusion, the combination of strong inhibitory dynamics and slow time-scale excitation decrease the possibility of rapid cascading firing events. Therefore, we have elected not to focus on our hybrid formalism in the present manuscript. However, in on-going work, we are indeed looking carefully at dynamical regimes where fast transient MFEs are more prevalent (e.g., highly excitatory network state where a particular set of neuron populations are poised to fire, thus generating spontaneous wave initiations and propagations) and examining their relevance to network dynamics and information coding.

4. There are other reductions two ODE-like systems, such as that Mattia & del Giudice. Although they were obtained by different methods, it is not clear to me that the approach describes here represents a major advance in the state-of-the-art.

**Response:** We thank the reviewer for informing us of the work by Mattia & del Giudice (Mattia et al., 2019; Mattia and Giudice, 2002), and have cited the references in the revised manuscript. The main result of Mattia & Giudice is an approach to the population density dynamics of networks with spiking neurons having non-Markovian membrane potentials. The effective one-dimensional description they obtained relied on both a mean-field approximation and the assumption of a relatively low synaptic noise. The authors then obtained the firing rate equation from the spectral expansion of the one-dimensional FP equation for the population density in the absence of synaptic filtering. Hence their firing rate equation can be considered as a spectral expansion of the FP operator. That is, they apply a spectral expansion to solve the FP equation. This is different from our moment equations, which derived from IF network to Master equation, FP equation, finally to a system of ODEs augmented with a maximum entropy principle to close the system. We note that even if there are synchronous network activities, our theoretical framework can still capture the dynamical feature of certain rapid transients. As mentioned above, we have checked that our network model of motion illusion operates in a dynamical regime where there are few or no MFEs. Therefore, we have elected not to focus on our hybrid formalism. However, in on-going work, we are indeed looking carefully at dynamical regimes where fast transient MFEs are more prevalent and examining their relevance to network dynamics.

Response to Reviewer # 3

The manuscript "Dimensional Reduction of Emergent Spatiotemporal Cortical Dynamics via a Maximum Entropy Moment Closure" employs a coarse-graining technique based on a population-density approach combined with an approximate moment closure to obtain a set of coarse-grained equations of motion for networks of spiking neurons. In particular, the authors here investigate the application of their method to large-scale models of V1 with realistic features in terms of connectivity. The manuscript is potentially an interesting contribution to the field. However, I have some major concerns about the employed approximations that need to be clarified before I would recommend publication in PloS CB.

1. The method proposed by the authors intends to perform a coarse-graining of leaky integrate-and-fire models. It rests on a maximum entropy closure of the moment hierarchy. To this end, the authors employ the equilibrium solution of the Fokker-Planck equation to find the expansion of the density in a number of moments that matches most closely the equilibrium distribution.

This scheme seems to be inconsistent with the with to obtain a coarse-grained theory for transient dynamics, because the equilibrium distribution, their eq. (22), is of course not a solution of the time-dependent problem.

The authors should explain how this approximation is justified or if I misunderstood their method

**Response:** We apologize for the poorly organized text and a typo that likely led to this misunderstanding. The instantaneous probability distribution and **not** the equilibrium distribution is used to define the voltage moments (typo in the original Eqn. (23)). We have previously shown that the maximum entropy closure is very effective in coarse-graining the network dynamics on the membrane leak time-scale. Also, in previous work, we showed that this framework can incorporate the transient synchronous firing of many neurons (as the so-called multiple firing events, or MFEs, (Zhang et al., 2014a; Zhang and Rangan, 2015; Zhang et al., 2019; Zhang et al., 2014b)). However, this involved a hybrid formalism, the partitioned ensemble average, which essentially uses the usual ensemble average (e.g., a Masters or a Fokker-Planck description (Cai et al., 2006)) between periods of fast synchronous spiking which have to be numerically sampled. In the present case, we checked that the large-scale I&F network model, in reproducing the essential features of motion illusion, is in a dynamical regime where there are few or no MFEs, therefore, we have elected not to focus on the hybrid formalism. However, in on-going work, we are indeed looking carefully at dynamical regimes where fast transient MFEs are more prevalent and examining their relevance to network dynamics and information coding.

We have rewritten this section in the text:

------ TEXT ------

Now we discuss the boundary conditions. Note that, $\rho_{j}^{Q}\left( v,t \right)=0$ when $\left( v\to-\infty\right)$ and the neuron can only fire by receiving an excitatory spike and cannot stream up to cross the voltage threshold. A negative flux at threshold $V_{T}$ is impossible and it can only reset once it arrives at the voltage threshold. Therefore, we have boundary conditions

$$\begin{aligned} \begin{aligned} \rho_{j}^{Q}\left( V_{T},t \right)= \rho_{j}^{Q}\left( -\infty,t \right)=0 \end{aligned}\#\left( 20 \right) \end{aligned}$$

We then consider a transient reset dynamics of the I&F type neurons in our model. Once the voltage crosses the voltage threshold $V_{T}$, it immediately resets to rest voltage $V_{R}$ without any refractory-period. Therefore, to ensure the continuity of voltage distribution, we have

$$\begin{aligned} \begin{aligned} J_{j}^{Q}\left[ \rho_{j}^{Q}\left( V_{T},t \right) \right]=J_{j}^{Q}\left[ \rho_{j}^{Q}\left( V_{R}^{+},t \right) \right]-J_{j}^{Q}\left[ \rho_{j}^{Q}\left( V_{R}^{-},t \right) \right] \end{aligned}\#\left( 21 \right) \end{aligned}$$

One of the most important statistical characterizations of neuronal networks is the firing rate, which reflects the suprathreshold network dynamics. The firing rate $m_{j}^{Q}\left( t \right)$ is related to the probability through the spiking threshold, which is given by

$$\begin{aligned} \begin{aligned} m_{j}^{Q}\left( t \right)= g_{L}J_{j}^{Q}\left[ \rho_{j}^{Q}\left( V_{T},t \right) \right]=-\frac{g_{L}\left( \sigma_{j}^{Q} \right)^{2}}{2}\partial_{v}\rho_{j}^{Q}\left( V_{T},t \right) \end{aligned}\#\left( 22 \right) \end{aligned}$$

under the boundary condition $\rho_{j}^{Q}\left( V_{T},t \right)=0$. And using the continuity of the voltage probability distribution, we also have

$$\begin{aligned} \begin{aligned} J_{j}^{Q}\left[ \rho_{j}^{Q}\left( V_{R}^{+},t \right) \right]-J_{j}^{Q}\left[ \rho_{j}^{Q}\left( V_{R}^{-},t \right) \right]=\frac{m_{j}^{Q}\left( t \right)}{g_{L}} \end{aligned}\#\left( 23 \right) \end{aligned}$$

In the evolution of this system, we can determine the firing rate $m_{j}^{Q}\left( t \right)$ via $\mathrm{Eq}\left( 22 \right)$once we know the solution of the full probability distribution function $\rho_{j}^{Q}\left( v,t \right)$. However, $\mathrm{Eqs}\left( 16-19 \right)$show that the voltage probability distribution function is specified with $\mu_{j}^{Q}$ and $\left( \sigma_{j}^{Q} \right)^{2}$, which are, in turn, functions of the firing rate $m_{i}^{Q}\left( t \right)$. Therefore, $\mathrm{Eq}\left( 16 \right)$ is a nonlinear partial differential equation.

As we showed previously, further dimensional reduction can be achieved (Zhang and Rangan, 2015).

First, let us define the $k^{th}$-order voltage moments $\chi_{j}^{Q,k}\left( t \right)$ of $\rho_{j}^{Q}\left( v,t \right)$ by:

$$\begin{aligned} \begin{aligned} \chi_{j}^{Q,k}\left( t \right)= \int_{-\infty}^{V^{T}} v^{k}\rho_{j}^{Q}\left( v,t \right)dv\#\# \end{aligned}\#\left( 24 \right) \end{aligned}$$

Combining $\mathrm{Eq}\left( 24 \right)$ and the original Fokker-Planck equation $\mathrm{Eq}\left( 16 \right)$, the evolution equations for the voltage moments $\chi_{j}^{Q,k}\left( t \right)$ become

$$\begin{aligned} \begin{aligned} \frac{d}{dt}\chi_{j}^{Q,k}\left( t \right)=-m_{j}^{Q}\left( t \right)-g_{L}\left[ \chi_{j}^{Q,k}-\mu_{j}^{Q} \right]\mathrm{for}k=1 \end{aligned}\#\left( 25 \right) \end{aligned}$$

$$\begin{aligned} \begin{aligned} \frac{d}{dt}\chi_{j}^{Q,k}\left( t \right)=-m_{j}^{Q}\left( t \right)V_{T}^{k}-kg_{L}\left[ \chi_{j}^{Q,k}-\mu_{j}^{Q}\chi_{j}^{Q,k-1}-\frac{k-1}{2}\left( \sigma_{j}^{Q} \right)^{2}\chi_{j}^{Q,k-2} \right]\mathrm{for}k>1 \end{aligned}\#\left( 26 \right) \end{aligned}$$

Now we obtain a system of ODEs ($Eq(25,26)$), but this system is not closed because we cannot directly compute the time-dependent firing rate $m_{j}^{Q}\left( t \right)$. Obviously, to evolve the system of voltage moments, we need to continuously update the firing rate at each time$t$. To compute the firing rate, we need to calculate the full voltage probability distribution $\rho_{j}^{Q}\left( v,t \right)$. However, given a finite set of moments $\left\{ \chi_{j}^{Q,k}\left( t \right) \right\}$, $k=1,2,\ldots,M$ and the terms $\mu_{j}^{Q}$ and $\left( \sigma_{j}^{Q} \right)^{2}$ on the right-hand-side of ODEs $Eq(25,26)$, we still cannot uniquely define the full voltage probability distribution. In general, there are many possible voltage distributions $\rho_{j}^{Q}\left( v,t \right)$ which are compatible with the entire information we have in, i.e.,$\left\{ \chi_{j}^{Q,k}\left( t \right) \right\}$, $\mu_{j}^{Q}$ and $\left( \sigma_{j}^{Q} \right)^{2}$. Thus we have a moment closure problem.

To deal with this, we need a way to compute the full probability distribution function $\rho_{j}^{Q}\left( v,t \right)$. From amongst all possibilities, we choose to use the one that lies closest to the equilibrium solution $\rho_{j,Eq}^{Q}\left( v \right)$ (see Appendix C.). Then, we apply a maximum entropy (ME) approach to find the solution and use an approximate maximum-entropy voltage distribution $\rho_{ME}\left( v,t \right)$ at each time $t$ to calculate the variables needed in temporal updates (more details in Appendix D.).

Based on general optimization theory, a necessary condition for probability distribution to be maximizer of the entropy maximization problem is that $\rho$ must satisfy all the constraints ($Eq (24-26)$) of the problem and the partial derivative of the Lagrangian function with respect to $\rho$ must also equal zero. To solve this problem, we first find the Lagrange multipliers $\boldsymbol{\lambda}_{\boldsymbol{j}}^{\boldsymbol{Q}}=\left[ \lambda_{j,0}^{Q}, \lambda_{j,1}^{Q}, \ldots,\lambda_{j,M}^{Q} \right]^{t}$ for each population, and then the classical solution of the distribution probability $\rho_{j}^{Q}\left( v,t \right)$ can be written as

$$\begin{aligned} \begin{aligned} \rho_{j}^{Q}\left( v,t \right)=\rho_{j,Eq}^{Q}\left( v \right)\exp\left( \lambda_{j,0}^{Q}-1 \right)exp\left( \sum_{k=1}^{M} \lambda_{j,k}^{Q}v^{k} \right) \end{aligned}\#\left( 27 \right) \end{aligned}$$

For convenience, we let $\lambda_{j,0}^{Q}=\lambda_{j,0}^{Q}-1$ and then

$$\begin{aligned} \begin{aligned} \rho_{j}^{Q}\left( v,t \right)=\rho_{j,Eq}^{Q}\left( v \right)exp\left( \sum_{k=0}^{M} \lambda_{j,k}^{Q}v^{k} \right) \end{aligned}\#\left( 28 \right) \end{aligned}$$

we can obtain the most possible distribution of $\rho_{j}^{Q}\left( v,t \right)$. Note the $Eq (22)$, we are now ready to use $\rho_{j}^{Q}\left( v,t \right)$ to solve the firing rate $m_{j}^{Q}\left( t \right)$ at time $t$

$$\begin{aligned} \begin{aligned} m_{j}^{Q}\left( t \right)= -\frac{g_{L}\left( \sigma_{j}^{Q} \right)^{2}}{2}\partial_{v}\rho_{j}^{Q}\left( V_{T},t \right)=\frac{g_{L}\sigma_{j}^{Q}}{2C}exp\left( \sum_{k=0}^{M} \lambda_{j,k}^{Q}V_{T}^{k} \right) \end{aligned}\#\left( 29 \right) \end{aligned}$$

where C is the normalization factor. Combine $Eq (25,26)$ with $Eq (29)$, the partial moment equations have a closure with the ME principle. We call this closed-form by the augmented ODE system. found that in the simulations we performed in this paper, good performance can be achieved with only 4 augmented variables $\left\{ \chi_{j}^{E,1}\left( t \right),\chi_{j}^{E,2}\left( t \right),\chi_{j}^{I,1}\left( t \right),\chi_{j}^{I,2}\left( t \right) \right\}$ for each CG patch. (A more complete and detailed discussion can be found in our previous work (Zhang et al., 2014a; Zhang and Rangan, 2015; Zhang et al., 2014b).)

**------**

2. I do not understand the rationale behind the approximation by the authors explained surrounding eq. (26). It is clear that a Gaussian approximation for the stationary membrane potential distribution is good as long as the mean input is sufficiently far from the threshold.

But I do not understand why they are using expressions (18) and (19) directly to compute the mean and variance of the equilibrium distribution by eq. (22), instead of using equations (24) and (25) for k = 1 and k = 2. Using the latter, they would correctly include the additional temporal filtering of the moments.

In their approach, the only temporal filtering seems to be coming from the slow synaptic currents.

The approximation by the authors, instead, seems to be identical to the Arrhenius approximation used in Brunel 2000, eq. 22 for the instantaneous firing rate.

**Response:** We apologize for this confusion. The Gaussian approximation of Eqn. (26) is **not** the same as the derivation of Eqn. (22), which is directly derived from Fokker-Planck equations Eqns. (16) and (17) using the drift and diffusion terms Eqns. (18) and (19). To address this confusion, we have revised the main text (see below) and have detailed the derivation of Eqn. (22) in a new Appendix C.

Thank you for observing that the approximation in Eqn. (26) is indeed identical to the Arrhenius approximation in Brunel 2000. In organizing this manuscript, we had hoped to provide a series of models that modelers can choose according to their particular needs. While the full theoretical framework yields a system of augmented ODEs, our moment closure (even in the case when only 2-4 moments are needed) involves a maximum entropy computation that can be non-trivial and costly to implement. Therefore, in the original manuscript we offered Eqn. (26), bypassing the maximum entropy calculation, and should allow for quick and dirty computations, especially when the phenomenon can be modeled by dynamics sufficiently far from spiking threshold.

We have added the following clarifying text

------ TEXT ------

We first focus on the evolving process of voltage probability distribution induced by various synaptic inputs. Therefore, we define the probability flux $J_{j}^{Q}\left[ \rho_{j}^{Q}\left( v,t \right) \right]$, which, conventionally, represents the probability crossing a voltage point $v$ at time point $t$ . In the Master equation $\mathrm{Eq}\left( 15 \right)$, there are one type of streaming flux due to the relaxation dynamics and another type of spike-driven flux induced by voltage jumps once receiving an input spike. Various researchers have utilized the Master equation combining with the probability flux to analytically or numerically solve the evolution of voltage probability density and treat the network dynamics (e.g., (De Kamps, 2003; Ly, 2013; Ly and Tranchina, 2007; Nykamp and Tranchina, 2000)).

For further simplification and theoretical analysis, we try to do some rational approximations. We assume that the voltage jumps due to the external Poisson spikes as well as the recurrent network spikes are small, so the Master equation $\mathrm{Eq}\left( 15 \right)$ can be approximated by a standard Fokker-Planck type equation,

$$\begin{aligned} \begin{aligned} \frac{d\rho_{j}^{Q}\left( v,t \right)}{dt}+g_{L}\partial_{v}J_{j}^{Q}\left[ \rho_{j}^{Q}\left( v,t \right) \right]=0, \mathrm{for} v\in\left[ V_{R},\left. V_{T} \right) \right. \end{aligned}\#\left( 16 \right) \end{aligned}$$

where the probability flux $J_{j}^{Q}\left[ \rho_{j}^{Q}\left( v,t \right) \right]$ is of a simpler form,

$$\begin{aligned} \begin{aligned} J_{j}^{Q}\left[ \rho_{j}^{Q}\left( v,t \right) \right]=-\left( v-\mu_{j}^{Q} \right)\rho_{j}^{Q}\left( v,t \right)-\frac{\left( \sigma_{j}^{Q} \right)^{2}}{2}\partial_{v}\rho_{j}^{Q}\left( v,t \right) \end{aligned}\#\left( 17 \right) \end{aligned}$$

with drift and diffusion terms. The drift and diffusion coefficients $\mu_{j}^{Q}$ (also called slaving-voltage) and $\sigma_{j}^{Q}$ depend on the spatiotemporal network couplings as well as cortical activities $m_{i}^{E/I}$, and can be written as

$$\mu_{j}^{Q}=\left( V_{L}g_{L}+ f_{j}^{Q}\eta_{j}^{Q}+N_{E}S_{fast}^{QE}\sum_{i} K_{fast}^{QE}\left( \left| c_{j}-c_{i} \right| \right)m_{i}^{E} \right.$$

$$\begin{aligned} \begin{aligned} \left. -N_{I}S_{fast}^{QI}\sum_{i} K_{fast}^{QI}\left( \left| c_{j}-c_{i} \right| \right)m_{i}^{I}+I_{j,slow}^{QE} \right)/{g_{L}} \end{aligned}\#\left( 18 \right) \end{aligned}$$

$$\left( \sigma_{j}^{Q} \right)^{2}=\left( \left( f_{j}^{Q} \right)^{2}\eta_{j}^{Q}+N_{E}\sum_{i} {\left( S_{fast}^{QE}K_{fast}^{QE}\left( \left| c_{j}-c_{i} \right| \right) \right)^{2}m}_{i}^{E} \right.$$

$$\begin{aligned} \begin{aligned} \left. + N_{I}\sum_{i} \left( S_{fast}^{QI}K_{fast}^{QI}\left( \left| c_{j}-c_{i} \right| \right) \right)^{2}m_{i}^{I} \right)/{g_{L}} \end{aligned}\#\left( 19 \right) \end{aligned}$$

The detailed derivations are in Appendix A, and some relevant references can be found in (Cai et al., 2006; Nykamp and Tranchina, 2000; Rangan et al., 2008; Zhang et al., 2014a; Zhang et al., 2014b).

------ APPENDIX ------

**Appendix C.**

We consider the Fokker-Planck equation

$$\begin{aligned} \begin{aligned} \frac{d\rho_{j}^{Q}\left( v,t \right)}{dt}+g_{L}\partial_{v}J_{j}^{Q}\left[ \rho_{j}^{Q}\left( v,t \right) \right]=0，\mathrm{for}v\in\left[ V_{R},\left. V_{T} \right) \right. \end{aligned}\#\left( 16 \right) \end{aligned}$$

and the flux

$$\begin{aligned} \begin{aligned} J_{j}^{Q}\left[ \rho_{j}^{Q}\left( v,t \right) \right]=-\left( v-\mu_{j}^{Q} \right)\rho_{j}^{Q}\left( v,t \right)-\frac{\left( \sigma_{j}^{Q} \right)^{2}}{2}\partial_{v}\rho_{j}^{Q}\left( v,t \right) \end{aligned}\#\left( 17 \right) \end{aligned}$$

with the boundary conditions

$$\begin{aligned} \begin{aligned} \rho_{j}^{Q}\left( V_{T},t \right)= \rho_{j}^{Q}\left( -\infty,t \right)=0 \end{aligned}\#\left( 20 \right) \end{aligned}$$

and the direct relation between firing rate and the probability flux

$$\begin{aligned} \begin{aligned} m_{j}^{Q}\left( t \right)= g_{L}J_{j}^{Q}\left[ \rho_{j}^{Q}\left( V_{T},t \right) \right]=-\frac{g_{L}\left( \sigma_{j}^{Q} \right)^{2}}{2}\partial_{v}\rho_{j}^{Q}\left( V_{T},t \right) \end{aligned}\#\left( 22 \right) \end{aligned}$$

Furthermore, we induce an equilibrium firing rate $\bar{m}_{j}^{Q}$ to solve the equilibrium probability distribution $\rho_{j,Eq}^{Q}\left( v \right)$. The derivation of the equilibrium solution $\rho_{j,Eq}^{Q}\left( v \right)$ is as follows,

$$\int_{v}^{V_{T}} \partial_{v}J_{j}^{Q}\left[ \rho_{j,Eq}^{Q}\left( v \right) \right]= J_{j}^{Q}\left[ \rho_{j,Eq}^{Q}\left( V_{T} \right) \right]-J_{j}^{Q}\left[ \rho_{j,Eq}^{Q}\left( v \right) \right]$$

$$\begin{aligned} \begin{aligned} =\frac{\bar{m}_{j}^{Q}}{g_{L}}+\left( v-\mu_{j}^{Q} \right)\rho_{j,Eq}^{Q}\left( v \right)+\frac{\left( \sigma_{j}^{Q} \right)^{2}}{2}\partial_{v}\rho_{j,Eq}^{Q}\left( v \right)=0 \end{aligned}\#\left( 39 \right) \end{aligned}$$

then, multiply with $exp\left[ \left( \frac{v-\mu_{j}^{Q}}{\sigma_{j}^{Q}} \right)^{2} \right]$, we obtain

$$\frac{2\bar{m}_{j}^{Q}}{g_{L}\left( \sigma_{j}^{Q} \right)^{2}}exp\left[ \left( \frac{v-\mu_{j}^{Q}}{\sigma_{j}^{Q}} \right)^{2} \right]=-\left[ \frac{2\left( v-\mu_{j}^{Q} \right)}{\left( \sigma_{j}^{Q} \right)^{2}}+\partial_{v} \right]\rho_{j,Eq}^{Q}\left( v \right)exp\left[ \left( \frac{v-\mu_{j}^{Q}}{\sigma_{j}^{Q}} \right)^{2} \right]$$

$$\begin{aligned} \begin{aligned} =-\partial_{v}\left( \rho_{j,Eq}^{Q}\left( v \right)exp\left[ \left( \frac{v-\mu_{j}^{Q}}{\sigma_{j}^{Q}} \right)^{2} \right] \right) \end{aligned}\#\left( 40 \right) \end{aligned}$$

let $s=\left( v-\mu_{j}^{Q} \right)/{\sigma_{j}^{Q}}$, change the variable and integrate$s$ from $\left( \max\left\{ v,V_{R} \right\}-\mu_{j}^{Q} \right)/{\sigma_{j}^{Q}}$ to $\left( V_{T}-\mu_{j}^{Q} \right)/{\sigma_{j}^{Q}}$ , we could obtain the equilibrium probability distribution

$$\begin{aligned} \begin{aligned} \rho_{j,Eq}^{Q}\left( v \right)=\frac{2\bar{m}_{j}^{Q}}{g_{L}\sigma_{j}^{Q}}exp\left[ \left( \frac{v-\mu_{j}^{Q}}{\sigma_{j}^{Q}} \right)^{2} \right]\int_{\left( max\left\{ v,V_{R} \right\}-\mu_{j}^{Q} \right)/{\sigma_{j}^{Q}}}^{\left( V_{T}-\mu_{j}^{Q} \right)/{\sigma_{j}^{Q}}} exp\left( s^{2} \right)ds \end{aligned}\#\left( 41 \right) \end{aligned}$$

------

3. I was missing a comparison of the methods used by the authors to the direct simulation of the spiking network. At least for some small examples that can still be simulated. Otherwise it is hard to see how good the made approximations are, in particular regarding my concerns about the correct treatment of the time-dependent behavior, raised above.

**Response:** Thank you for this helpful suggestion. In our previous paper (Zhang et al., 2019), we have compare some of the important statistical dynamical features, e.g., first order (firing rates and voltage distribution) and high-order statistics (such as multiple firing events and synchrony), between the LIF network, and the corresponding Masters and FP equations. For the comparison of probability densities, one can see Figure 3 in (Zhang et al., 2019). (Figure included below.) For further comparison and performance of our reduction, please refer to the following (Zhang and Rangan, 2015; Zhang et al., 2019), in which we compare in detail the Master equation or FP equation with our moment closure method in simulating a variety of dynamical regimes, including homogeneous and synchronous network activities. For homogeneous networks activity, our reduction can capture the firing rate and density distribution almost exactly, and for synchronous network activity, it can faithfully capture highly heterogeneous neuronal network dynamics such as multiple firing events (or MFEs; please see Figure 4 in (Zhang and Rangan, 2015), also included below).

Figure 3 in (Zhang et al., 2019) A nearly homogeneous scenario for our PEA-framework. In panels-A and B we compare the steady-state voltage distributions for $\rho^{E}\left( v \right)$ and $\rho^{I}\left( v \right)$, respectively. The red line is achieved by IF model and the green dash line by the Master equation. In panels C and D we compare the steady-state voltage distributions for $\rho^{E}\left( v \right)$ and $\rho^{E}\left( v \right)$ respectively. The red line is achieved by IF equation and the blue line by Fokker-Planck equation.

Figure 4 in (Zhang and Rangan, 2015) Panel-a illustrates the dynamics of this IF-network; Panel-b and Panel-c are similar to Panel-a, except that they illustrate the system-trajectories corresponding to master equation and our moment model (derive from FP equation), respectively. The feedforward input to this regime is of higher rate and lower strength, and thus is more like a mean current input. This input, in conjunction with a stronger recurrent excitation and weaker recurrent inhibition (differ from the parameter regime in the condition of motion illusion), causes this regime to generate MFEs more frequently; this regime generates frequent large MFEs with a characteristic frequency close to 32 Hz.

Minor points:

1. Eq. (26) is not correctly normalized. Factor 2 missing in the pre-factor. Response: We have corrected this.

$$\begin{aligned} \begin{aligned} \tilde{\rho}_{j,Eq}^{Q}\left( v \right)\approx\frac{1}{\sqrt{\pi}\sigma_{j}^{Q}}exp\left( -\left( \frac{v-\mu_{j}^{Q}}{\sigma_{j}^{Q}} \right)^{2} \right) \end{aligned} \end{aligned}$$

2. The maximum entropy approach should be explained more explicitly in the methods part.3. Response: We have added this in **Appendix D.**
3. Typos: the index i has not limited to labeling single neuron Response: We have corrected this.

$$\begin{aligned} \begin{aligned} I_{lgn}^{j}= C\epsilon\left[ 1+\frac{1}{2}\left( 1+\cos2\left( \theta_{j}-\theta_{t} \right) \right)\sin\left( \omega t\boldsymbol{-}\varphi_{j} \right) \right] \end{aligned}\#\left( 5 \right) \end{aligned}$$

note that here the index $j$ has not limited to labeling single neuron

4. eq. (22) time dependence t on left side should be v same in eq. (26) Response: We have corrected this.

References

Cai, D., Tao, L., Rangan, A.V., and Mclaughlin, D.W. (2006). KINETIC THEORY FOR NEURONAL NETWORK DYNAMICS. Communications in Mathematical Sciences *4*, 97-127.

Cai, D., Tao, L., Shelley, M.J., and Mclaughlin, D.W. (2004). An effective kinetic representation of fluctuation-driven neuronal networks with application to simple and complex cells in visual cortex. Proceedings of the National Academy of Sciences of the United States of America *101*, 7757-7762.

De Kamps, M. (2003). A simple and stable numerical solution for the population density equation. Neural Computation *15*, 2129-2146.

Deco, G., and Rolls, E.T. (2005). Attention, short-term memory, and action selection: A unifying theory. Progress in Neurobiology *76*, 236-256.

Fusi, S. (2003). Spike-driven synaptic plasticity for learning correlated patterns of mean firing rates. Reviews in The Neurosciences *14*, 73.

Gerstner, W. (2000). Population Dynamics of Spiking Neurons: Fast Transients, Asynchronous States, and Locking. Neural Computation *12*, 43-89.

Haskell, E., Nykamp, D.Q., and Tranchina, D. (2001). Population density methods for large-scale modelling of neuronal networks with realistic synaptic kinetics: cutting the dimension down to size. Network: Computation In Neural Systems *12*, 141-174.

Ly, C. (2013). A principled dimension-reduction method for the population density approach to modeling networks of neurons with synaptic dynamics. Neural Computation *25*, 2682-2708.

Ly, C., and Tranchina, D. (2007). Critical Analysis of Dimension Reduction by a Moment Closure Method in a Population Density Approach to Neural Network Modeling. Neural Computation *19*, 2032-2092.

Mattia, M., Biggio, M., Galluzzi, A., and Storace, M. (2019). Dimensional reduction in networks of non-Markovian spiking neurons: Equivalence of synaptic filtering and heterogeneous propagation delays. PLOS Computational Biology *15*.

Mattia, M., and Giudice, P.D. (2002). Population dynamics of interacting spiking neurons. Physical Review E *66*, 051917.

Nykamp, D.Q., and Tranchina, D. (2000). A population density approach that facilitates large-scale modeling of neural networks: analysis and an application to orientation tuning. Journal of Computational Neuroscience *8*, 19-50.

Rangan, A.V., Kovacic, G., and Cai, D. (2008). Kinetic theory for neuronal networks with fast and slow excitatory conductances driven by the same spike train. Physical Review E *77*, 041915.

Rekauzke, X.S., Nortmann, N., Staadt, X.R., Hock, H.S., Schoner, X.G., and Jancke, D. (2016). Temporal Asymmetry in Dark–Bright Processing Initiates Propagating Activity across Primary Visual Cortex. The Journal of Neuroscience *36*, 1902-1913.

Wong, K.F., and Wang, X. (2006). A Recurrent Network Mechanism of Time Integration in Perceptual Decisions. The Journal of Neuroscience *26*, 1314-1328.

Zhang, J., Newhall, K.A., Zhou, D., and Rangan, A.V. (2014a). Distribution of correlated spiking events in a population-based approach for Integrate-and-Fire networks. Journal of Computational Neuroscience *36*, 279-295.

Zhang, J., and Rangan, A.V. (2015). A reduction for spiking integrate-and-fire network dynamics ranging from homogeneity to synchrony. Journal of Computational Neuroscience *38*, 355-404.

Zhang, J., Shao, Y., Rangan, A.V., and Tao, L. (2019). A coarse-graining framework for spiking neuronal networks: from strongly-coupled conductance-based integrate-and-fire neurons to augmented systems of ODEs. Journal of Computational Neuroscience *46*, 211-232.

Zhang, J., Zhou, D., Cai, D., and Rangan, A.V. (2014b). A coarse-grained framework for spiking neuronal networks: between homogeneity and synchrony. Journal of Computational Neuroscience *37*, 81-104.
